# A Promising Food-Coaching Intervention Program to Achieve Optimal Gestational Weight Gain in Overweight and Obese Pregnant Women: Pilot Randomized Controlled Trial of a Smartphone App

**DOI:** 10.2196/13013

**Published:** 2019-10-24

**Authors:** Ling-Jun Li, Izzuddin M Aris, Wee Meng Han, Kok Hian Tan

**Affiliations:** 1 Department of Obstetrics & Gynecology KK Women's and Children's Hospital Singapore Singapore; 2 Division of Chronic Disease Research Across the Lifecourse Department of Population Medicine Harvard Medical School and Harvard Pilgrim Health Care Institute Boston, MA United States; 3 Department of Nutrition KK Women's and Children's Hospital Singapore Singapore

**Keywords:** overweight, obesity, pregnant women, gestational weight gain, food diary, randomized controlled trial, smartphone app, food coaching, dietary recommendation, feasibility

## Abstract

**Background:**

Traditional dietary recommendations for achieving optimal gestational weight gain are ineffective for pregnant women due to the lack of real-time communication and tedious consultation processes.

**Objective:**

In this pilot study, we aimed to determine the feasibility of a novel food-coaching smartphone app for controlling gestational weight gain and macronutrient intake among overweight and obese pregnant women.

**Methods:**

We designed a randomized controlled trial and recruited 30 overweight and obese pregnant women (1:1 ratio) during 18-20 weeks of gestation and followed them up after 4 and 8 weeks, respectively. Both groups received standard pregnancy dietary orientation at recruitment, while the intervention group received 8 weeks of real-time food coaching via a smartphone app. This food-coaching smartphone app (Glycoleap, Holmusk, Singapore) aimed to improve care and outcomes for people with diabetes. Pregnant women using this app were able to upload food images (eg, a picture of a meal, a drink, or a dessert) and received real-time and detailed food-coaching comments and guidance provided by professional dietitians during the day (8 AM to 8 PM). We recorded detailed characteristics during recruitment and examined anthropometry at all visits. We compared the mean differences of the 8-week gestational weight gain and macronutrient intake between the two groups.

**Results:**

Upon study completion, three subjects dropped out from the intervention, and one gave birth prematurely in the control group. The acceptance rate of the smartphone app was 90%. More participants achieved optimal gestational weight gain per week in the intervention group (8/12, 67%) than in the control group (5/14, 36%). After the 8-week intervention, women in the intervention group appeared to have lower gestational weight gain (mean difference=–0.08 kg; 95% CI –1.80 to 1.63) and cholesterol intake (mean difference=–31.73 mg; 95% CI –102.91 to 39.45) than those in the control group.

**Conclusions:**

Our findings showed that this food-coaching smartphone app is feasible and favorable for weight gain control and cholesterol intake control among overweight and obese pregnant women. Although our results were not significant (perhaps, attributed to the small sample size), it provided proof of concept for the feasibility of applying such technology in future randomized controlled trials with a larger sample size, an earlier intervention onset, and a longer follow-up for overweight and obese pregnant women.

## Introduction

Overweight and obese pregnant women are often at an increased risk of a series of maternal and offspring adverse outcomes [1-3]. In developed countries, overweight and obese women accounted for 30%-50% of fertile women [4-6]. Therefore, the high prevalence of overweight and obesity among pregnant women signifies a substantial burden to public health welfare worldwide. Current clinical care management of overweight and obese women during pregnancy consists of information on healthy eating practices according to standard dietary guidelines both worldwide [7-10] and in Singapore [11,12], in terms of restricting daily energy intake; balancing the proportion of complex carbohydrate (33%-40%), protein (20%), and fat (40%); and lowering cholesterol intake However, the reported effects were equivocal due to limitations such as low compliance, delayed feedback, late initiation, and inefficient delivery of intervention [13,14].

In recent years, the use of mobile technology (such as smartphone apps) for patient care has been increasing. The feasibility and efficacy of such technology has been proven for weight management among pediatric obese patients [15] and for glucose control in mothers with gestational diabetes mellitus during pregnancy [16]. Furthermore, a systematic review summarized 12 studies using phone**-**based reporting interventions such as video call, phone calls, short messaging service (SMS) and smartphone apps and showed consistent evidence that although such approaches could help pregnant women control their gestational weight gain, they were not effective in preventing other pregnancy outcomes such as gestational diabetes mellitus [17]. Another systematic review of four randomized controlled trials (RCTs) did not draw any firm conclusions on the effects of mobile app interventions during pregnancy on maternal knowledge, behavior change, and perinatal health outcomes due to heterogeneity of interventions, comparators, and outcome measures across all RCTs [18]. In addition, based on the intervention evaluation alone, none of these mobile technologies provide a real-time communication between end users and medical workers or dietitians, thus likely affecting the compliance and efficacy of the phone-based intervention. Given the high prevalence of smartphone app usage among pregnant women, more rigorous studies are needed to optimize the study design and implementation of these technologies in improving maternal health outcomes. In this pilot randomized controlled trial study, we tested a food-coaching intervention program delivered through a smartphone app in overweight and obese pregnant subjects without gestational diabetes mellitus and examined its feasibility, acceptance, and preliminary utility during an 8-week follow-up in second trimester.

## Methods

### Study Design and Population

We conducted a prospective, two-arm, unblinded RCT in a subsidized clinic within a tertiary government hospital in Singapore (KK Women’s and Children’s Hospital [KKH]) between March and July 2018. We recruited pregnant women if they were Singapore citizens or permanent residents, overweight or obese (ie, prepregnancy or booking body mass index≥25 kg/m^2^), age≥21 years, between 18 and 20 weeks’ gestation at the time of recruitment, planning to deliver in KKH, capable of reading and writing in English, and able to download and use the smartphone app. We excluded women with special dietary restrictions due to medical conditions such as type 1 or type 2 diabetes, gestational diabetes mellitus, hypertension, and chronic kidney disease.

We conducted the study according to the tenets of the Declaration of Helsinki and obtained approval by the SingHealth Centralized Institutional Review Board and the National Health Group’s Domain Specific Review Board. We obtained written informed consent from all pregnant women at baseline recruitment. As the primary aim of this study is to test the feasibility of the smartphone app in the pilot phase, we only obtained the local institutional review board approval (CIRB 2017/2132), but did not register for an online RCT number.

### Randomization and Procedures

**Figure 1:**
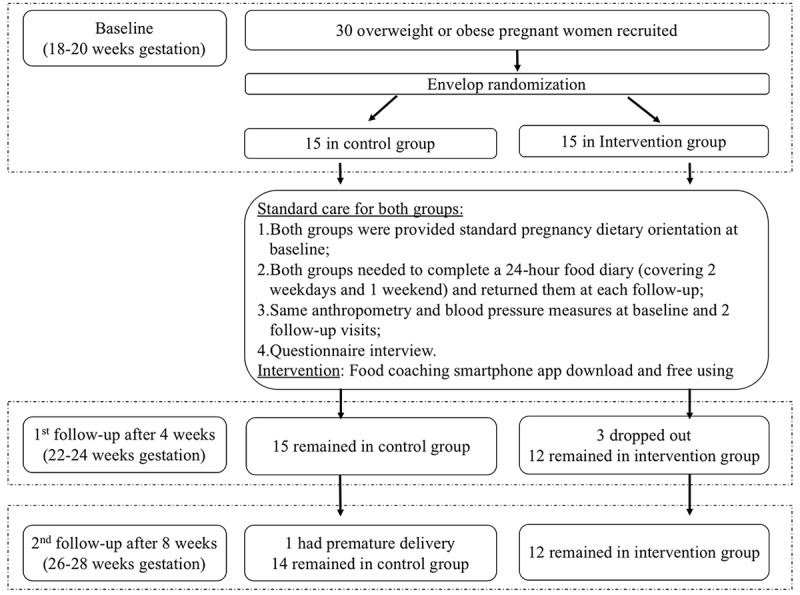
Study flow of the randomized controlled trial.

Research coordinators randomly assigned pregnant women to the intervention (food-coaching smartphone app) or control group (standard pregnancy dietary orientation) in a 1:1 allocation ratio, using the envelope randomization method (Figure 1).

#### Standard Pregnancy Dietary Orientation

The same research coordinators administered the standard dietary orientation to women in the intervention and control groups using standardized materials, including basic nutrition principles of choosing a wide variety of nutrient-dense foods, limiting intake of high-fat and high-sugar food and beverages, and guiding food portion sizes, with reference to the local gestational dietary guidelines by the Health Promotion Board [19]. We informed participants in both groups about the recommended gestational weight gain, together with some simple physical activity pointers.

#### A Food-Coaching Smartphone App

The intervention group participants could download and use the food-coaching smartphone app (Glycoleap, Holmusk, Singapore) for free for up to 8 weeks during the RCT. The app aimed to improve care and outcomes of people with type 2 diabetes in terms of diet control. Pregnant women using this app were able to upload food images and received real-time and detailed food-coaching comments and guidance from professional dietitians during the day (8 AM to 8 PM). According to the local pregnancy guideline [20], food dietitians rated the food image from 1 to 5 (1=the least recommended score, 5=the most recommended scored) and provided feedback in terms of degree and balance of the food items and composition (Multimedia Appendix 1). Although this smartphone app was available on both Apple and Google store platforms, we did not think there was a high chance for the controls to obtain access to this app for two reasons: (1) This smartphone app requires an in-store purchase of up to SG $30 (US $22) per month, which is not subsidized by any local Singaporean medical insurance. All participants were recruited from the government tertiary hospital’s subsidized clinic; therefore, the chances for them to pay from their own pocket to afford additional medical care are low. (2) Only pregnant women randomized into the intervention group were informed of the name and company of the smartphone app, while those in the control group were not provided any information regarding the app. In addition, subjects in the intervention and control groups did not know of each other.

We recruited all subjects at baseline (18-20 weeks’ gestation) and followed them up after 4 weeks (22-24 weeks’ gestation) and 8 weeks (26-28 weeks’ gestation).

### Outcomes

Based on the usage of the food-coaching smartphone app, we assessed the feasibility of the smartphone app by collecting participant feedback in the intervention group, using an evaluation form at the 8-week follow-up visit (Multimedia Appendix 2). In addition, we assessed the compliance in the intervention group via the times of log in for each participant in the first half and second half of the follow-up. We calculated the proportion of participants with optimal second trimester gestational weight gain based on the Institute of Medicine guidelines for overweight (0.23-0.33 kg/week) and obese women (0.17-0.27 kg/week) [9,21]. We assisted all pregnant subjects in quantifying their food and beverage intake. Such methods of assessing dietary data have been widely published [22-24]. We obtained data on 24-hour food recall at recruitment and collected self-administered 3-day food diary data (Multimedia Appendix 3) in both groups at two follow-ups. We analyzed the dietary records using an online nutrient analysis software [25], which was derived from locally available foods [20,26,27]. The Singapore Health Promotion Board online guidelines provided specific dietary instructions upon completion of the 3-day food diary and provided a picture compendium to each subject to facilitate identification of each type and quantity of food consumed. The compendium of food pictures consisted of photographs of individual food items coded to reflect the portion size of food items and standardize the estimated amounts eaten. The guidelines also used standard bowls, glasses, and spoons of varying sizes to estimate the volume of fluids or the amount of food consumed. Research coordinators entered each recorded food item into an online nutrient analysis database (Food Information and Nutrient Database system, Health Promotion Board, Singapore) based on a food composition database of locally available foods [25]. The daily food intake data were then summed up, and the total daily energy, macronutrients (carbohydrate, fiber, fat, and protein), calcium, cholesterol, and sodium intakes were averaged over each 3-day period and listed in kilocalories, grams, and percentages in the data summary form. The intake was compared between the intervention and control groups at the two follow-up visits.

### Statistical Analysis

We applied the Fisher exact test and Student *t* test for categorical and continuous variables, respectively, to compare characteristics between the intervention and control groups. We used linear regression to examine the effect of the food-coaching smartphone app on weight gain control and macronutrient intake between two groups. Mean differences as an estimate referencing the control group were shown in linear regression. We performed statistical analysis using STATA (Version 14.0. STATA Corp, College Station, Texas), set a two-tailed *P* value for significance at .05, and provided the 95% CIs for all estimates.

## Results

Among 30 pregnant women recruited at baseline with 1:1 allocation, 26 (12 in the intervention group and 14 in the control group) completed the 8-week RCT. The detailed RCT flow chart is shown in Figure 1. Based on the log-in records, the uptake of this smartphone app among our intervention subjects was up to 90% at the beginning (n=15) and 70% at the end of the study (n=12), respectively. Based on the 12 returned user evaluation forms, 75% (n=9) of the app users found that the app was easy to operate, more than 80% (n=10) thought the food-coaching guidance was acceptably fast, and 90% (n=11) were satisfied and reported that the app had somewhat or greatly improved their own diet*.* Therefore, they would recommend the smartphone app to family and friends. Furthermore, according to the backlog records from the smartphone app provider, we assessed compliance by calculating the average log-in frequency in the first and second half of the 8-week follow-up. Each of the 12 subjects in the intervention group logged into the smartphone app 8 times per week in the first 4 weeks, on an average, but this number reduced to 2 times per week in the subsequent 4 weeks.

Tables 1-3 show the baseline and follow-up characteristics between the intervention and control groups. More participants met the Institute of Medicine recommendation for optimal gestational weight gain per week in the intervention group (4-week follow-up: 7/12, 58%; 8-week follow-up: 8/12, 67%) than in the control group (4-week follow-up: 8/15, 53%; 8-week follow-up: 5/14, 36%).

**Table 1:** Comparison of baseline characteristics between the intervention and control groups (baseline recruitment at 18-20 weeks’ gestation).

Characteristics		Intervention group (n=15)	Control group (n=15)	*P* value^a^
Age (years), mean (SD)		29.3 (4.4)	30.7 (5)	.45
**Ethnicity, n (%)**				.15
	Chinese	2 (13)	6 (40)	
	Malay	12 (80)	8 (53)	
	Indian	1 (7)	0 (0)	
	Others	0 (0)	1 (6)	
Smoking history (yes), n (%)		4 (27)	1 (6)	.33
**Parity, n (%)**				.60
	0	7 (47)	5 (33)	
	1	4 (27)	7 (47)	
	≥2	4 (27)	3 (20)	
Maternal college degree (yes), n (%)		3 (20)	5 (33)	.68
Paternal college degree (yes), n (%)		1 (7)	4 (27)	.33
Household income ≥US $6000/month, n (%)		3 (20)	6 (40)	.43
**Past history of pregnancy outcomes**				.16
	Gestational diabetes mellitus, n (%)	0 (0)	3 (20)	
	Hypertensive disorders during pregnancy, n (%)	0 (0)	1 (7)	
	Prepregnancy weight (kg), mean (SD)	78.5 (15.8)	73.9 (6.8)	.31
	Prepregnancy body mass index (kg/m^2^), mean (SD)	31.0 (4.7)	29.4 (2.2)	.27
Gestational age at recruitment (weeks), mean (SD)		19.2 (2.1)	17.6 (2.8)	.08
Baseline body mass index (kg/m^2^), mean (SD)		34.2 (4.3)	31.3 (2.8)	.04
Baseline systolic blood pressure (mm Hg), mean (SD)		118.6 (8.9)	116.8 (9.4)	.59
Baseline diastolic blood pressure (mm Hg), mean (SD)		69.4 (4.6)	64.1 (7.9)	.03

^a^Student *t* test or Fisher exact test.

**Table 2:** Comparison of follow-up measures between the intervention and control groups (4-week follow-up at 22-24 weeks’ gestation).

Clinical measures			Intervention group (n=12)		Control group (n=15)		*P* value^a^
**Anthropometric and blood pressure measures, mean (SD)**							
	First follow-up body mass index (kg/m^2^)	36.4 (4.9)		31.9 (2.6)		.003	
	First follow-up systolic blood pressure (mm Hg)	121.9 (6.8)		120.9 (6.4)		.73	
	First follow-up diastolic blood pressure (mm Hg)	70.9 (7.5)		66.5 (6.7)		.15	
	Weight gain from baseline (kg)	1.3 (1.5)		1.5 (1.6)		.83	
Patients obtaining optimal weight gain per week, n (%)			7 (58)		8 (53)		.67
**Dietary measures, mean (SD)**							
	Energy intake (kcal)	1370.5 (359.4)		1514.0 (362.6)		.32	
	Carbohydrate (g)	177.5 (55.3)		189.5 (49.3)		.56	
	Protein (g)	52.6 (15.0)		65.1 (17.5)		.06	
	Total fat (g)	49.4 (15.9)		54.3 (17.3)		.46	
	Cholesterol (mg)	182.0 (90.6)		246.9 (109.7)		.11	
	Calcium (g)	513.4 (320.8)		513.3 (286.9)		.99	
	Dietary fiber (g)	12.9 (4.3)		12.9 (3.5)		.95	
	Sodium (g)	2.4 (0.8)		2.8 (0.8)		.18	

^a^Student *t* test, Fisher exact test, or Wilcoxon signed rank test.

**Table 3:** Comparison of follow-up measures between the intervention and control groups (8-week follow-up at 26-28 weeks’ gestation).

Clinical measures			Intervention group (n=12)		Control group (n=14)		*P* value^a^
**Anthropometric and blood pressure measures, mean (SD)**							
	Second follow-up body mass index (kg/m^2^)	36.0 (4.6)		32.4 (2.8)		.02	
	Second follow-up systolic blood pressure (mm Hg)	120.2 (7.5)		117.3 (7.3)		.33	
	Second follow-up diastolic blood pressure (mm Hg)	69.6 (9.1)		67.4 (5.8)		.47	
	Weight gain from baseline (kg)	2.9 (1.9)		3.0 (2.24)		.92	
Patients obtaining optimal weight gain per week, n (%)			8 (67)		5 (36)		.43
**Dietary measures, mean (SD)**							
	Energy intake (kcal)	1370.5 (359.4)		1514.0 (362.6)		.44	
	Carbohydrate (g)	1815.0)		170.6 (49.4)		.61	
	Protein (g)	56.7 (19.8)		58.6 (15.8)		.79	
	Total fat (g)	54.3 (20.7)		43.5 (17.5)		.15	
	Cholesterol (mg)	196.2 (89.3)		220.6 (87.9)		.48	
	Calcium (g)	461.0 (246.0)		489.1 (222.9)		.76	
	Dietary fiber (g)	13.0 (5.3)		12.9 (4.5)		.99	
	Sodium (g)	2.2 (0.7)		2.4 (0.8)		.55	

^a^Student *t* test, Fisher exact test, or Wilcoxon signed rank test.

Although not significant, we found a trend among women in the intervention group who tended to have less weight gain than those in the control group at the 4-week follow-up (mean difference=–0.15 kg; 95% CI –1.51 to 1.21) and 8-week follow-up (mean difference=–0.08 kg; –1.80 to 1.63; Table 4). In addition, women in the intervention group tended to consume less cholesterol than those in the control group at the 4-week follow-up (mean difference=–64.87 mg; 95% CI –146.04 to 16.31) and 8-week follow-up (mean difference=–31.73 mg; 95% CI –102.91 to 39.45).

**Table 4:** Weight gain from baseline and dietary intake between the intervention and control groups.

Dietary components	Mean difference between intervention and control (ref) groups			
	4-week follow-up (22-24 weeks’ gestation)^a^		8-week follow-up (26-28 weeks’ gestation)^b^	
	β (95% CI)	*P* value	β (95% CI)	*P* value
Weight gain from baseline (kg)	–0.15 (–1.51 to 1.21)	.83	–0.08 (–1.80 to 1.63)	.92
Energy intake (kcal)	–143.55 (–431.66 to 144.56)	.32	123.99 (–222.74 to 470.72)	.47
Carbo (g)	–12.05 (–53.54 to 29.44)	.56	9.35 (–33.62 to 52.31)	.66
Protein (g)	–12.58 (–25.69 to 0.53)	.06	–2.08 (–16.73 to 12.57)	.77
Total fat (g)	–4.91 (–18.24 to 8.42)	.46	11.11 (–4.60 to 26.81)	.16
Cholesterol (mg)	–64.87 (–146.04 to 16.31)	.11	–31.73 (–102.91 to 39.45)	.37
Calcium (g)	0.04 (–241.08 to 241.16)	>.99	–19.16 (–211.31 to 172.99)	.84
Dietary fiber (g)	0.10 (–2.97 to 3.17)	.95	0.20 (–3.82 to 4.22)	.92
Sodium (g)	–413.16 (–1032.09 to 205.76)	.18	–98.83 (–692.65 to 494.98)	.73

^a^Intervention group: n=12; control group: n=15.

^b^Intervention group: n=12; control group: n=14.

## Discussion

In this in a small sample RCT, we used a smartphone app to guide overweight and obese pregnant women to eat heathier in order to obtain optimal weight gain. We noted a high uptake of the smartphone app, as described above, and found evidence that pregnant women in the intervention group were more likely to have optimal gestational weight gain and consume less cholesterol compared with women in the control group. Although our pilot results were not significant, it provided proof of concept for the feasibility of applying such technology in future RCTs with a larger sample size, an earlier intervention onset, and a longer follow-up for overweight and obese pregnant women.

Although the dietary intervention has been proven to be effective in terms of weight gain control among overweight and obese pregnant women [28,29], there are huge variations in the delivery of dietary recommendation such as timing of intervention initiation, intensity of intervention, and feedback availability [13,28-31]. In a recently published systematic review summarizing 12 phone-based intervention studies on gestational weight gain control outcomes, most of the studies used phone calls or SMS to provide weight control guidelines, encouraged physical activity, and provided educational information about healthy nutrition. Several studies suggested that telephone communication is one of the most cost-effective tools to keep track of pregnant women’s health [32]. However, phone-based interventions are typically initiated by health care providers and might not effectively motivate pregnant women to self-manage their behaviors. Given the high prevalence of smartphone app usage among pregnant women to improve their healthy behaviors, no firm conclusion has been drawn in terms of improving perinatal outcomes [18]. One of the major drawbacks is the lack of rigorous studies examining self-managing and self-regulatory behaviors in pregnant women. Our food-coaching app is more flexible and interactive and has greater variety of communication modalities, thus overcoming all aforementioned limitations in current technology. Given the high smartphone usage rate (up to 80%) among Singaporeans [33] and high prevalence (up to 30%) of overweight and obesity among pregnant women in tertiary hospital settings [4], use of a mobile app to promote healthy dietary intake is feasible and likely a promising intervention strategy. Our findings showed feasibility and acceptability of such a food-coaching smartphone app. For example, 75% of the app users found that the app was easy to operate, and more than 80% thought the food-coaching guidance was acceptably fast. Furthermore, 90% reported that the app had somewhat or greatly improved their own diet*.*

Interestingly, we did observe a trend of lower energy, carbohydrate, protein, total fat, and cholesterol intake in the first 4 weeks of the intervention, while most of the macronutrients did not maintain the same trend at 8 weeks of follow-up, except for cholesterol. This may be because pregnant subjects were more inclined to remember food items that are high in cholesterol (ie, saturated fat, red meat, full-fat dairy products) and were able to make an effort to avoid such food items compared with others. However, further studies are needed to verify our findings, as such findings with a small sample might be biased. Although the effect estimates are in the desired direction and supported the proof of concept as the primary focus in this pilot RCT, we still need to further verify the utility of such a smartphone app in a larger targeted population with an earlier phase intervention during pregnancy and a longer follow-up.

The strength of our study included a prospective RCT study design and the use of standard protocols in anthropometric measures and dietary assessments. However, our study has significant limitations regarding the loss to follow-up and a small sample for analysis.

In conclusion, our study provides proof of concept that smartphone technology is feasible and acceptable in clinical dietary guidance among overweight and obese pregnant women. In the future, we will adopt this food-coaching smartphone app and test its utility in a larger setting with a targeted population, earlier intervention, and longer follow-up throughout pregnancy.

## Appendix

Multimedia Appendix 1 The electronic interface layout of the food-coaching smartphone app (Glycoleap).

Multimedia Appendix 2 Self-evaluation of acceptability on the GlycoLeap application during pregnancy.

Multimedia Appendix 3 Food diary form.

Multimedia Appendix 4 CONSORT‐EHEALTH checklist (V 1.6.1).

## Abbreviations

KKH: KK Women’s and Children’s Hospital
RCT: randomized controlled trial
SMS: short messaging service
