# Supplementary figures and images for: A Promising Food-Coaching Intervention Program to Achieve Optimal Gestational Weight Gain in Overweight and Obese Pregnant Women: Pilot Randomized Controlled Trial of a Smartphone App
Source: JMIR Form Res. 2019 Oct 24;3(4):e13013. doi: 10.2196/13013 (PMC6914273; doi:10.2196/13013)

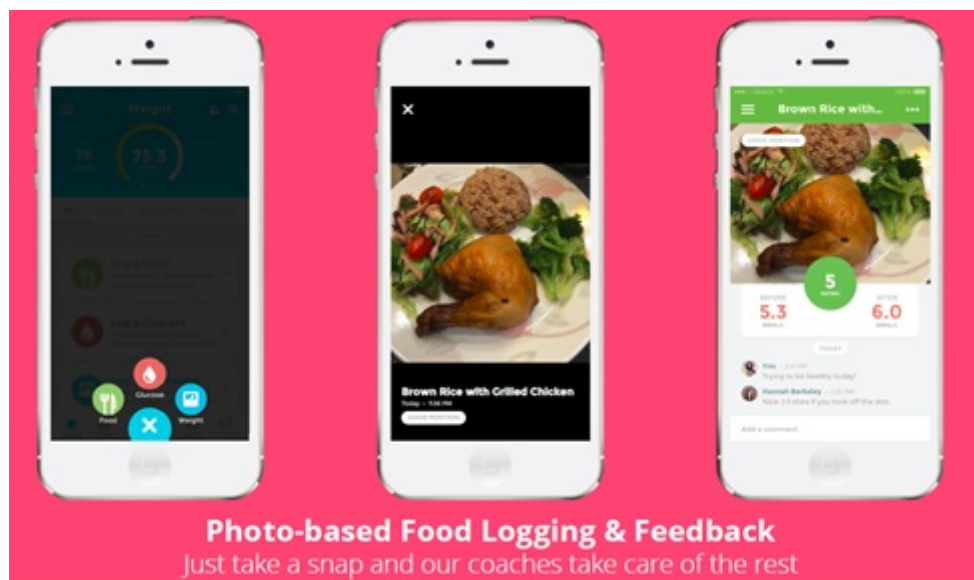

Supplement: Multimedia Appendix 1 [file formative_v3i4e13013_app1.pdf]
