# Supplementary material for: A Promising Food-Coaching Intervention Program to Achieve Optimal Gestational Weight Gain in Overweight and Obese Pregnant Women: Pilot Randomized Controlled Trial of a Smartphone App
Source: JMIR Form Res. 2019 Oct 24;3(4):e13013. doi: 10.2196/13013 (PMC6914273; doi:10.2196/13013)
Supplement: Multimedia Appendix 2 [file formative_v3i4e13013_app2.pdf]

Self-evaluation on Acceptability on GlycoLeap Application during Pregnancy

1. How often did you log into glycoLeap?
    a) Less than once a week;
    b) About once a week;
    c) About 2-3 times a week;
    d) About 4-5 times a week;
    e) Every day.
2. How often did you upload photos into glycoLeap?
    a) Everyday;
    b) Once a wekk;
    c) About 2-3 times a week;
    d) About 4-5 times a week;
    e) Irregular.
3. How easy was it to upload a food picture to glycoLeap?
    a) Very difficult
    b) Somehow difficult
    c) Somehow easy
    d) Very easy
4. Have you used some application like glycoLeap to monitor your diet before?
    a) No
    b) Yes

    If Yes, what was/were those application(s) that you have used before? Which one do you think is better?

    ____
5. How easy was it to interpret the food coach's comments or feedback?
    a) Very difficult
    b) Somehow difficult
    c) Somehow easy
    d) Very easy
6. Do you think it is acceptably fast to receive a food coach's comments or feedback during the daytime 8am-8pm?
    a) Yes
    b) No

    If No, why?

    ____
7. Have you made any changes to your diet after using glycoLeap?
    a) No
    b) Yes

    If Yes, in what way that you tried to change?

    ____

8. Have you ever viewed your steps counts in the GlycoLeap mobile app?
    a) Never
    b) Yes, but not often
    c) Yes, very often
9. Are you satisfied with the food imaging coaching on GlycoLeap?
    a) Not satisfied
    b) Slightly satisfied.
    c) Quite satisfied.
    d) Very satisfied.
10. Do you think it is useful to supervise your own diet through glycoleap?
    a) No
    b) Yes, but not much
    c) Yes, significantly
11. Do you find it easier to manage your food choices during pregnancy after using GlycoLeap?
    a) No
    b) Yes, but not much
    c) Yes, significantly
12. Have you ever talked to your family or friends about glycoleap?
    a) No
    b) Yes
13. How will you recommend GlycoLeap to your friends or family?
    a) Will not recommend
    b) Probably will recommend
    c) Quite likely to recommend
    d) Will highly recommend
14. Will you be willing to pay for such service like GlycoLeap in the future?
    a) No
    b) Yes
15. Do you think you have enough support from your husband via using GlycoLeap?
    a) No
    b) Yes

    If No, why?

    ____
16. Do you think there has been a significant help in changing your diet with your husband's involvement via using GlycoLeap?
    a) No
    b) Yes

    If No, why?

    ____
