# Supplementary material for: A Promising Food-Coaching Intervention Program to Achieve Optimal Gestational Weight Gain in Overweight and Obese Pregnant Women: Pilot Randomized Controlled Trial of a Smartphone App
Source: JMIR Form Res. 2019 Oct 24;3(4):e13013. doi: 10.2196/13013 (PMC6914273; doi:10.2196/13013)
Supplement: Multimedia Appendix 3 [file formative_v3i4e13013_app3.pdf]

Reg No 198904227G

KK Women's and Children's Hospital
SingHealth

Patient's name label

Food Diary Form

(Date: ____________________)

- ***Please record all the foods, drinks and snacks that you usually consume.***
- ***Use ONE form for each day and record your intake on 2 weekdays and 1 weekend.***
- *Include the quantity as accurately as possible, using the food pictures provided in the booklet to estimate your food portion sizes.*

MEAL	FOOD (Description, cooking method, quantity)<br><br>For example: Plain rice, A1; Chicken, without skin, stir-fried with soy sauce, C2; broccoli, boiled, J2; 1 small red apple, with skin	DRINKS (Description, quantity)<br><br>For example: Coffee with or without milk or sugar; type of milk (non-fat, low fat); instant, ground, decaffeinated; 250ml.
**Breakfast (_____)**		
**Mid – morning (_____)**		
**Lunch (_____)**		
**Mid – afternoon (_____)**		
**Dinner (_____)**		

**Supper (_____)**		
**Others (include any other snacks or drinks consume d during the day)**		
