# Supplementary material for: A Promising Food-Coaching Intervention Program to Achieve Optimal Gestational Weight Gain in Overweight and Obese Pregnant Women: Pilot Randomized Controlled Trial of a Smartphone App
Source: JMIR Form Res. 2019 Oct 24;3(4):e13013. doi: 10.2196/13013 (PMC6914273; doi:10.2196/13013)
Supplement: Multimedia Appendix 4 [file formative_v3i4e13013_app4.pdf]

CONSORT-EHEALTH (V 1.6.1) - Submission/Publication Form

The CONSORT-EHEALTH checklist is intended for authors of randomized trials evaluating web-based and Internet-based applications/interventions, including mobile interventions, electronic games (incl multiplayer games), social media, certain telehealth applications, and other interactive and/or networked electronic applications. Some of the items (e.g. all subitems under item 5 - description of the intervention) may also be applicable for other study designs.

The goal of the CONSORT EHEALTH checklist and guideline is to be
a) a guide for reporting for authors of RCTs,
b) to form a basis for appraisal of an ehealth trial (in terms of validity)

CONSORT-EHEALTH items/subitems are MANDATORY reporting items for studies published in the Journal of Medical Internet Research and other journals / scientific societies endorsing the checklist.

Items numbered 1., 2., 3., 4a., 4b etc are original CONSORT or CONSORT-NPT (non-pharmacologic treatment) items.
Items with Roman numerals (i., ii, iii, iv etc.) are CONSORT-EHEALTH extensions/clarifications.

As the CONSORT-EHEALTH checklist is still considered in a formative stage, we would ask that you also RATE ON A SCALE OF 1-5 how important/useful you feel each item is FOR THE PURPOSE OF THE CHECKLIST and reporting guideline (optional).

Mandatory reporting items are marked with a red *.
In the textboxes, either copy & paste the relevant sections from your manuscript into this form - please include any quotes from your manuscript in QUOTATION MARKS,
or answer directly by providing additional information not in the manuscript, or elaborating on why the item was not relevant for this study.

YOUR ANSWERS WILL BE PUBLISHED AS A SUPPLEMENTARY FILE TO YOUR PUBLICATION IN JMIR AND ARE CONSIDERED PART OF YOUR PUBLICATION (IF ACCEPTED).
Please fill in these questions diligently. Information will not be copyedited, so please use proper spelling and grammar, use correct capitalization, and avoid abbreviations.

DO NOT FORGET TO SAVE AS PDF _AND_ CLICK THE SUBMIT BUTTON SO YOUR ANSWERS ARE IN OUR DATABASE !!!

Citation Suggestion (if you append the pdf as Appendix we suggest to cite this paper in the caption):
Eysenbach G, CONSORT-EHEALTH Group
CONSORT-EHEALTH: Improving and Standardizing Evaluation Reports of Web-based and Mobile Health Interventions
J Med Internet Res 2011;13(4):e126
URL: http://www.jmir.org/2011/4/e126/
doi: 10.2196/jmir.1923
PMID: 22209829

* Required

Your name *

First Last

Lingjun Li

Primary Affiliation (short), City, Country *

University of Toronto, Toronto, Canada

Singapore

Your e-mail address *

abc@gmail.com

queenie.li.l@kkh.com.sg

Title of your manuscript *

Provide the (draft) title of your manuscript.

A Promising Food-Coaching Intervention Program to Obtain Optimal Gestational Weight Gain in Pregnant Overweight and Obese Women: A Pilot Randomized Control Trial of A Smartphone App

Name of your App/Software/Intervention *

If there is a short and a long/alternate name, write the short name first and add the long name in brackets.

Glycoleap

Evaluated Version (if any)

e.g. "V1", "Release 2017-03-01", "Version 2.0.27913"

N.A.

Language(s) *

What language is the intervention/app in? If multiple languages are available, separate by comma (e.g. "English, French")

English

URL of your Intervention Website or App

e.g. a direct link to the mobile app on app in appstore (itunes, Google Play), or URL of the website. If the intervention is a DVD or hardware, you can also link to an Amazon page.

https://glycoleap.com/

URL of an image/screenshot (optional)

https://www.apkmonk.com/app/com.holr

Accessibility *

Can an enduser access the intervention presently?

- ○ access is free and open
- ○ access only for special usergroups, not open
- ◉ access is open to everyone, but requires payment/subscription/in-app purchases
- ○ app/intervention no longer accessible
- ○ Other:

Primary Medical Indication/Disease/Condition *

e.g. "Stress", "Diabetes", or define the target group in brackets after the condition, e.g. "Autism (Parents of children with)", "Alzheimers (Informal Caregivers of)"

Overweight and obese pregnant women

Primary Outcomes measured in trial *

comma-separated list of primary outcomes reported in the trial

weight gain and macronutrient intake duri

Secondary/other outcomes

Are there any other outcomes the intervention is expected to affect?

Your answer

Recommended "Dose" *

What do the instructions for users say on how often the app should be used?

- (●) Approximately Daily
- ( ) Approximately Weekly
- ( ) Approximately Monthly
- ( ) Approximately Yearly
- ( ) "as needed"
- ( ) Other:

Approx. Percentage of Users (starters) still using the app as recommended after 3 months *

- (selected) unknown / not evaluated
- 0-10%
- 11-20%
- 21-30%
- 31-40%
- 41-50%
- 51-60%
- 61-70%
- 71%-80%
- 81-90%
- 91-100%
- Other:

Overall, was the app/intervention effective? *

- yes: all primary outcomes were significantly better in intervention group vs control
- partly: SOME primary outcomes were significantly better in intervention group vs control
- no statistically significant difference between control and intervention
- potentially harmful: control was significantly better than intervention in one or more outcomes
- inconclusive: more research is needed
- (selected) Other: No significant difference between control and intervention, yet this is a [illegible]

Article Preparation Status/Stage *

At which stage in your article preparation are you currently (at the time you fill in this form)

- ○ not submitted yet - in early draft status
- ◉ not submitted yet - in late draft status, just before submission
- ○ submitted to a journal but not reviewed yet
- ○ submitted to a journal and after receiving initial reviewer comments
- ○ submitted to a journal and accepted, but not published yet
- ○ published
- ○ Other:

Journal *

If you already know where you will submit this paper (or if it is already submitted), please provide the journal name (if it is not JMIR, provide the journal name under "other")

- ○ not submitted yet / unclear where I will submit this
- ○ Journal of Medical Internet Research (JMIR)
- ◉ JMIR mHealth and UHealth
- ○ JMIR Serious Games
- ○ JMIR Mental Health
- ○ JMIR Public Health
- ○ JMIR Formative Research
- ○ Other JMIR sister journal
- ○ Other:

Is this a full powered effectiveness trial or a pilot/feasibility trial? *

- ◉ Pilot/feasibility
- ○ Fully powered

Manuscript tracking number *

If this is a JMIR submission, please provide the manuscript tracking number under "other" (The ms tracking number can be found in the submission acknowledgement email, or when you login as author in JMIR. If the paper is already published in JMIR, then the ms tracking number is the four-digit number at the end of the DOI, to be found at the bottom of each published article in JMIR)

- (selected) no ms number (yet) / not (yet) submitted to / published in JMIR
- Other:

TITLE AND ABSTRACT

1a) TITLE: Identification as a randomized trial in the title

1a) Does your paper address CONSORT item 1a? *

I.e does the title contain the phrase "Randomized Controlled Trial"? (if not, explain the reason under "other")

- (selected) yes
- Other:

1a-i) Identify the mode of delivery in the title

Identify the mode of delivery. Preferably use “web-based” and/or “mobile” and/or “electronic game” in the title. Avoid ambiguous terms like “online”, “virtual”, “interactive”. Use “Internet-based” only if Intervention includes non-web-based Internet components (e.g. email), use “computer-based” or “electronic” only if offline products are used. Use “virtual” only in the context of “virtual reality” (3-D worlds). Use “online” only in the context of “online support groups”. Complement or substitute product names with broader terms for the class of products (such as “mobile” or “smart phone” instead of “iphone”), especially if the application runs on different platforms.

	1	2	3	4	5	
subitem not at all important	○	○	○	○	●	essential

Does your paper address subitem 1a-i? *

Copy and paste relevant sections from manuscript title (include quotes in quotation marks "like this" to indicate direct quotes from your manuscript), or elaborate on this item by providing additional information not in the ms, or briefly explain why the item is not applicable/relevant for your study

Yes, we used smartphone app in the title.
"A Promising Food-Coaching Intervention Program to Obtain Optimal Gestational Weight Gain in Pregnant Overweight and Obese Women: A Pilot Randomized Control Trial of A Smartphone App"

1a-ii) Non-web-based components or important co-interventions in title

Mention non-web-based components or important co-interventions in title, if any (e.g., “with telephone support”).

	1	2	3	4	5	
subitem not at all important	◉	○	○	○	○	essential

Does your paper address subitem 1a-ii?

Copy and paste relevant sections from manuscript title (include quotes in quotation marks "like this" to indicate direct quotes from your manuscript), or elaborate on this item by providing additional information not in the ms, or briefly explain why the item is not applicable/relevant for your study

We do not have any non-web-based component.

1a-iii) Primary condition or target group in the title

Mention primary condition or target group in the title, if any (e.g., “for children with Type I Diabetes”)
Example: A Web-based and Mobile Intervention with Telephone Support for Children with Type I Diabetes: Randomized Controlled Trial

	1	2	3	4	5	
subitem not at all important	○	○	○	○	◉	essential

Does your paper address subitem 1a-iii? *

Copy and paste relevant sections from manuscript title (include quotes in quotation marks "like this" to indicate direct quotes from your manuscript), or elaborate on this item by providing additional information not in the ms, or briefly explain why the item is not applicable/relevant for your study

Yes, it is for overweight and obese pregnant women.
"A Promising Food-Coaching Intervention Program to Obtain Optimal Gestational Weight Gain in Pregnant Overweight and Obese Women: A Pilot Randomized Control Trial of A Smartphone App"

1b) ABSTRACT: Structured summary of trial design, methods, results, and conclusions

NPT extension: Description of experimental treatment, comparator, care providers, centers, and blinding status.

1b-i) Key features/functionalities/components of the intervention and comparator in the METHODS section of the ABSTRACT

Mention key features/functionalities/components of the intervention and comparator in the abstract. If possible, also mention theories and principles used for designing the site. Keep in mind the needs of systematic reviewers and indexers by including important synonyms. (Note: Only report in the abstract what the main paper is reporting. If this information is missing from the main body of text, consider adding it)

	1	2	3	4	5	
subitem not at all important	○	○	○	○	◉	essential

Does your paper address subitem 1b-i? *

Copy and paste relevant sections from the manuscript abstract (include quotes in quotation marks "like this" to indicate direct quotes from your manuscript), or elaborate on this item by providing additional information not in the ms, or briefly explain why the item is not applicable/relevant for your study

Yes, we did.
"Both groups received standard pregnancy dietary orientation at recruitment, while intervention group received an 8-week's real-time food coaching via a smartphone app."

1b-ii) Level of human involvement in the METHODS section of the ABSTRACT

Clarify the level of human involvement in the abstract, e.g., use phrases like “fully automated” vs. “therapist/nurse/care provider/physician-assisted” (mention number and expertise of providers involved, if any). (Note: Only report in the abstract what the main paper is reporting. If this information is missing from the main body of text, consider adding it)

	1	2	3	4	5	
subitem not at all important	○	○	○	○	◉	essential

Does your paper address subitem 1b-ii?

Copy and paste relevant sections from the manuscript abstract (include quotes in quotation marks "like this" to indicate direct quotes from your manuscript), or elaborate on this item by providing additional information not in the ms, or briefly explain why the item is not applicable/relevant for your study

It is a registered nutritionist or dietitian-based food coaching smartphone app. And it has been addressed in the Methods.
"Pregnant women using this app were able to upload food images (e.g., a picture of a meal, a drink, or a dessert) and received real-time and detailed food coaching comments and guidance provided by professional dietitians during the day (8am-8pm)."

1b-iii) Open vs. closed, web-based (self-assessment) vs. face-to-face assessments in the METHODS section of the ABSTRACT

Mention how participants were recruited (online vs. offline), e.g., from an open access website or from a clinic or a closed online user group (closed usergroup trial), and clarify if this was a purely web-based trial, or there were face-to-face components (as part of the intervention or for assessment). Clearly say if outcomes were self-assessed through questionnaires (as common in web-based trials). Note: In traditional offline trials, an open trial (open-label trial) is a type of clinical trial in which both the researchers and participants know which treatment is being administered. To avoid confusion, use “blinded” or “unblinded” to indicated the level of blinding instead of “open”, as “open” in web-based trials usually refers to “open access” (i.e. participants can self-enrol). (Note: Only report in the abstract what the main paper is reporting. If this information is missing from the main body of text, consider adding it)

	1	2	3	4	5	
subitem not at all important	○	○	○	○	◉	essential

Does your paper address subitem 1b-iii?

Copy and paste relevant sections from the manuscript abstract (include quotes in quotation marks "like this" to indicate direct quotes from your manuscript), or elaborate on this item by providing additional information not in the ms, or briefly explain why the item is not applicable/relevant for your study

It is open, meaning the user and the coach interacted with each other throughout the smartphone app.
"Pregnant women using this app were able to upload food images (e.g., a picture of a meal, a drink, or a dessert) and received real-time and detailed food coaching comments and guidance provided by professional dietitians during the day (8am-8pm). "

1b-iv) RESULTS section in abstract must contain use data

Report number of participants enrolled/assessed in each group, the use/uptake of the intervention (e.g., attrition/adherence metrics, use over time, number of logins etc.), in addition to primary/secondary outcomes. (Note: Only report in the abstract what the main paper is reporting. If this information is missing from the main body of text, consider adding it)

	1	2	3	4	5	
subitem not at all important	○	○	○	○	◉	essential

Does your paper address subitem 1b-iv?

Copy and paste relevant sections from the manuscript abstract (include quotes in quotation marks "like this" to indicate direct quotes from your manuscript), or elaborate on this item by providing additional information not in the ms, or briefly explain why the item is not applicable/relevant for your study

Yes, we did.
"Upon study completion, 3 subjects dropped out from intervention while 1 gave birth prematurely from control group. The acceptance of smartphone app is 90%. More achieved optimal WG per week in intervention group (7/12, 58.3%) than in control group (5/14, 35.7%). Food coaching smartphone app seemed to help in reducing GWG and cholesterol intake."

1b-v) CONCLUSIONS/DISCUSSION in abstract for negative trials

Conclusions/Discussions in abstract for negative trials: Discuss the primary outcome - if the trial is negative (primary outcome not changed), and the intervention was not used, discuss whether negative results are attributable to lack of uptake and discuss reasons. (Note: Only report in the abstract what the main paper is reporting. If this information is missing from the main body of text, consider adding it)

	1	2	3	4	5	
subitem not at all important	○	○	○	○	◉	essential

Does your paper address subitem 1b-v?

Copy and paste relevant sections from the manuscript abstract (include quotes in quotation marks "like this" to indicate direct quotes from your manuscript), or elaborate on this item by providing additional information not in the ms, or briefly explain why the item is not applicable/relevant for your study

Yes, the primary outcome falls in the right direction yet without significance. It is due to the small samples that we recruited. However, the proof-of-concept is established and we have addressed all strength and limitation in our Discussion.
"Although our results were not significant (which could be attributed to the small sample size), it provided a proof-of-concept on the feasibility of applying such technology in future RCTs with a larger sample size, an earlier intervention onset, and a longer follow-up for overweight and obese pregnant women."

INTRODUCTION

2a) In INTRODUCTION: Scientific background and explanation of rationale

2a-i) Problem and the type of system/solution

Describe the problem and the type of system/solution that is object of the study: intended as stand-alone intervention vs. incorporated in broader health care program? Intended for a particular patient population? Goals of the intervention, e.g., being more cost-effective to other interventions, replace or complement other solutions? (Note: Details about the intervention are provided in “Methods” under 5)

	1	2	3	4	5	
subitem not at all important	○	○	○	○	◉	essential

Does your paper address subitem 2a-i? *

Copy and paste relevant sections from the manuscript (include quotes in quotation marks "like this" to indicate direct quotes from your manuscript), or elaborate on this item by providing additional information not in the ms, or briefly explain why the item is not applicable/relevant for your study

Yes.
"Therefore, the high prevalence of overweight and obesity among pregnant women signifies a substantial burden to public health welfare worldwide."
"Limitations in current intervention included: 1) Delayed feedback in dietary recommendation; 2) Interventions commenced during the mid-trimester when it is too late to avoid extra weight gain during pregnancy; 3) Lacking a useful interactive system to deliver the dietary recommendations conveniently. In addition, these limitations probably impacted adversely on participants' self-motivation and therefore, a majority of these interventions yielded unsatisfactory compliance rates (20~60%) [3]."

2a-ii) Scientific background, rationale: What is known about the (type of) system

Scientific background, rationale: What is known about the (type of) system that is the object of the study (be sure to discuss the use of similar systems for other conditions/diagnoses, if appropiate), motivation for the study, i.e. what are the reasons for and what is the context for this specific study, from which stakeholder viewpoint is the study performed, potential impact of findings [2]. Briefly justify the choice of the comparator.

	1	2	3	4	5	
subitem not at all important	○	○	○	○	◉	essential

Does your paper address subitem 2a-ii? *

Copy and paste relevant sections from the manuscript (include quotes in quotation marks "like this" to indicate direct quotes from your manuscript), or elaborate on this item by providing additional information not in the ms, or briefly explain why the item is not applicable/relevant for your study

Yes.
"In recent years, smartphone applications for daily monitoring of blood glucose levels and motivating physical activity has been tested in diabetic patients. The feasibility and efficacy of such technology has been proven in weight management among pediatric obese patients [16], and glucose control in mothers with GDM during pregnancy [17]."

2b) In INTRODUCTION: Specific objectives or hypotheses

Does your paper address CONSORT subitem 2b? *

Copy and paste relevant sections from the manuscript (include quotes in quotation marks "like this" to indicate direct quotes from your manuscript), or elaborate on this item by providing additional information not in the ms, or briefly explain why the item is not applicable/relevant for your study

Yes, we have provided a specific objective and hypothesis.
"In this pilot randomized controlled trial study, we tested a food-coaching intervention program in overweight and obese pregnant subjects using a smartphone application, and examined its feasibility, acceptance and preliminary utility during an 8 weeks' follow-up in 2nd trimester. We hypothesized that providing dietary recommendations through a smartphone application in a targeted pregnant population during pregnancy was feasible and acceptable and would have beneficial effects on weight gain control and dietary intake improvement. "

METHODS

3a) Description of trial design (such as parallel, factorial) including allocation ratio

Does your paper address CONSORT subitem 3a? *

Copy and paste relevant sections from the manuscript (include quotes in quotation marks "like this" to indicate direct quotes from your manuscript), or elaborate on this item by providing additional information not in the ms, or briefly explain why the item is not applicable/relevant for your study

Yes, together with a flow chart (Figure 1).
"We randomly assigned pregnant women to intervention group (using food coaching smartphone app) or to control group (receiving standard pregnancy dietary orientation) in a 1:1 allocation ratio, using the envelopment randomization method. Pregnant women, investigators and study staff were not masked to group allocation (Figure 1)."

3b) Important changes to methods after trial commencement (such as eligibility criteria), with reasons

Does your paper address CONSORT subitem 3b? *

Copy and paste relevant sections from the manuscript (include quotes in quotation marks "like this" to indicate direct quotes from your manuscript), or elaborate on this item by providing additional information not in the ms, or briefly explain why the item is not applicable/relevant for your study

Yes, we did mention.
"We recruited pregnant women if they fulfilled the following criteria : 1) being either Singapore citizens or permanent residents; 2) being overweight and obese with pre-pregnancy or booking BMI at 25 kg/m2 and above; 3) Aged 21 years and above; 4) between 18 and 20 weeks of gestation at the time of recruitment; 5) Planning to deliver in KKH; 6) Being capable of reading and writing in English; and 7) Having a smartphone and being able to download and use the smartphone app. We excluded women with special dietary restrictions due to medical conditions such as Type 1 or Type 2 diabetes, hypertension and chronic kidney disease."

3b-i) Bug fixes, Downtimes, Content Changes

Bug fixes, Downtimes, Content Changes: ehealth systems are often dynamic systems. A description of changes to methods therefore also includes important changes made on the intervention or comparator during the trial (e.g., major bug fixes or changes in the functionality or content) (5-iii) and other “unexpected events” that may have influenced study design such as staff changes, system failures/downtimes, etc. [2].

	1	2	3	4	5	
subitem not at all important	◉	○	○	○	○	essential

Does your paper address subitem 3b-i?

Copy and paste relevant sections from the manuscript (include quotes in quotation marks "like this" to indicate direct quotes from your manuscript), or elaborate on this item by providing additional information not in the ms, or briefly explain why the item is not applicable/relevant for your study

We conducted a survey to report technical problems like this. But it is not important in our paper.

4a) Eligibility criteria for participants

Does your paper address CONSORT subitem 4a? *

Copy and paste relevant sections from the manuscript (include quotes in quotation marks "like this" to indicate direct quotes from your manuscript), or elaborate on this item by providing additional information not in the ms, or briefly explain why the item is not applicable/relevant for your study

Yes.
"We recruited pregnant women if they fulfilled the following criteria : 1) being either Singapore citizens or permanent residents; 2) being overweight and obese with pre-pregnancy or booking BMI at 25 kg/m2 and above; 3) Aged 21 years and above; 4) between 18 and 20 weeks of gestation at the time of recruitment; 5) Planning to deliver in KKH; 6) Being capable of reading and writing in English; and 7) Having a smartphone and being able to download and use the smartphone app. We excluded women with special dietary restrictions due to medical conditions such as Type 1 or Type 2 diabetes, hypertension and chronic kidney disease."

4a-i) Computer / Internet literacy

Computer / Internet literacy is often an implicit “de facto” eligibility criterion - this should be explicitly clarified.

	1	2	3	4	5	
subitem not at all important	○	○	○	○	◉	essential

Does your paper address subitem 4a-i?

Copy and paste relevant sections from the manuscript (include quotes in quotation marks "like this" to indicate direct quotes from your manuscript), or elaborate on this item by providing additional information not in the ms, or briefly explain why the item is not applicable/relevant for your study

Yes, internet access is one of the inclusion criteria.
"7) Having a smartphone and being able to download and use the smartphone app."

4a-ii) Open vs. closed, web-based vs. face-to-face assessments:

Open vs. closed, web-based vs. face-to-face assessments: Mention how participants were recruited (online vs. offline), e.g., from an open access website or from a clinic, and clarify if this was a purely web-based trial, or there were face-to-face components (as part of the intervention or for assessment), i.e., to what degree got the study team to know the participant. In online-only trials, clarify if participants were quasi-anonymous and whether having multiple identities was possible or whether technical or logistical measures (e.g., cookies, email confirmation, phone calls) were used to detect/prevent these.

	1	2	3	4	5	
subitem not at all important	○	○	○	○	◉	essential

Does your paper address subitem 4a-ii? *

Copy and paste relevant sections from the manuscript (include quotes in quotation marks "like this" to indicate direct quotes from your manuscript), or elaborate on this item by providing additional information not in the ms, or briefly explain why the item is not applicable/relevant for your study

Yes.
"2. Food coaching smartphone app—Intervention group participants received a free download and use of a food coaching smartphone app (Glycoleap, Holmusk, Singapore) up to 8 weeks during the RCT."
"According to the local pregnancy guideline [19], food dietitians rated the food image from 1 to 5 (1 as the least recommended score while 5 as the most recommended scored) and gave feedback in terms of degree and balance of the food items and composition."

4a-iii) Information giving during recruitment

Information given during recruitment. Specify how participants were briefed for recruitment and in the informed consent procedures (e.g., publish the informed consent documentation as appendix, see also item X26), as this information may have an effect on user self-selection, user expectation and may also bias results.

	1	2	3	4	5	
subitem not at all important	○	○	○	○	◉	essential

Does your paper address subitem 4a-iii?

Copy and paste relevant sections from the manuscript (include quotes in quotation marks "like this" to indicate direct quotes from your manuscript), or elaborate on this item by providing additional information not in the ms, or briefly explain why the item is not applicable/relevant for your study

Yes.
"According to the local pregnancy guideline [19], food dietitians rated the food image from 1 to 5 (1 as the least recommended score while 5 as the most recommended scored) and gave feedback in terms of degree and balance of the food items and composition"

4b) Settings and locations where the data were collected

Does your paper address CONSORT subitem 4b? *

Copy and paste relevant sections from the manuscript (include quotes in quotation marks "like this" to indicate direct quotes from your manuscript), or elaborate on this item by providing additional information not in the ms, or briefly explain why the item is not applicable/relevant for your study

Yes, in the antenatal clinic of KK Women's and Children's Hospital.
"We conducted a prospective, two-arm (n=30) and unblinded randomized controlled trial (RCT) in a subsidized clinic within a tertiary government hospital in Singapore (KK Women's and Children's Hospital; KKH)."

4b-i) Report if outcomes were (self-)assessed through online questionnaires

Clearly report if outcomes were (self-)assessed through online questionnaires (as common in web-based trials) or otherwise.

	1	2	3	4	5	
subitem not at all important	●	○	○	○	○	essential

Does your paper address subitem 4b-i? *

Copy and paste relevant sections from the manuscript (include quotes in quotation marks "like this" to indicate direct quotes from your manuscript), or elaborate on this item by providing additional information not in the ms, or briefly explain why the item is not applicable/relevant for your study

No, it is not applicable in our study design.

4b-ii) Report how institutional affiliations are displayed

Report how institutional affiliations are displayed to potential participants [on ehealth media], as affiliations with prestigious hospitals or universities may affect volunteer rates, use, and reactions with regards to an intervention.(Not a required item – describe only if this may bias results)

	1	2	3	4	5	
subitem not at all important	(selected)	○	○	○	○	essential

Does your paper address subitem 4b-ii?

Copy and paste relevant sections from the manuscript (include quotes in quotation marks "like this" to indicate direct quotes from your manuscript), or elaborate on this item by providing additional information not in the ms, or briefly explain why the item is not applicable/relevant for your study

We did mention the hospital yet we do not think it is very important at the pilot stage.

5) The interventions for each group with sufficient details to allow replication, including how and when they were actually administered

5-i) Mention names, credential, affiliations of the developers, sponsors, and owners

Mention names, credential, affiliations of the developers, sponsors, and owners [6] (if authors/evaluators are owners or developer of the software, this needs to be declared in a “Conflict of interest” section or mentioned elsewhere in the manuscript).

	1	2	3	4	5	
subitem not at all important	○	(selected)	○	○	○	essential

Does your paper address subitem 5-i?

Copy and paste relevant sections from the manuscript (include quotes in quotation marks "like this" to indicate direct quotes from your manuscript), or elaborate on this item by providing additional information not in the ms, or briefly explain why the item is not applicable/relevant for your study

Yes we did mention the developer yet it is not important in our study since the credential would not affect the recruitment rate or our study conduction. "Intervention group participants received a free download and use of a food coaching smartphone app (Glycoleap, Holmusk, Singapore) up to 8 weeks during the RCT."

5-ii) Describe the history/development process

Describe the history/development process of the application and previous formative evaluations (e.g., focus groups, usability testing), as these will have an impact on adoption/use rates and help with interpreting results.

	1	2	3	4	5	
subitem not at all important	(selected)	○	○	○	○	essential

Does your paper address subitem 5-ii?

Copy and paste relevant sections from the manuscript (include quotes in quotation marks "like this" to indicate direct quotes from your manuscript), or elaborate on this item by providing additional information not in the ms, or briefly explain why the item is not applicable/relevant for your study

We did mention the application was used in chronic diabetic patients, and we adopted it for pregnant women. Yet the backend food coaches are experienced with dietary intake during pregnancy as well.

5-iii) Revisions and updating

Revisions and updating. Clearly mention the date and/or version number of the application/intervention (and comparator, if applicable) evaluated, or describe whether the intervention underwent major changes during the evaluation process, or whether the development and/or content was “frozen” during the trial. Describe dynamic components such as news feeds or changing content which may have an impact on the replicability of the intervention (for unexpected events see item 3b).

	1	2	3	4	5	
subitem not at all important	(selected)	○	○	○	○	essential

Does your paper address subitem 5-iii?

Copy and paste relevant sections from the manuscript (include quotes in quotation marks "like this" to indicate direct quotes from your manuscript), or elaborate on this item by providing additional information not in the ms, or briefly explain why the item is not applicable/relevant for your study

No, this is not important in our paper.

5-iv) Quality assurance methods

Provide information on quality assurance methods to ensure accuracy and quality of information provided [1], if applicable.

	1	2	3	4	5	
subitem not at all important	○	○	○	◉	○	essential

Does your paper address subitem 5-iv?

Copy and paste relevant sections from the manuscript (include quotes in quotation marks "like this" to indicate direct quotes from your manuscript), or elaborate on this item by providing additional information not in the ms, or briefly explain why the item is not applicable/relevant for your study

Yes, the log-in information and also the verification dietary intake has been mentioned.
"According to the local pregnancy guideline [19], food dietitians rated the food image from 1 to 5 (1 as the least recommended score while 5 as the most recommended scored) and gave feedback in terms of degree and balance of the food items and composition."
"We used weekend diary to validate the dietary data obtained from the weekday diary."
"Based on the usage of the food coaching smartphone app, we encouraged all pregnant women in intervention group to fill up the user evaluation form at the 8-week follow-up visit (Supplementary Figure 2), to assess the feasibility and collect participants' feedback."

5-v) Ensure replicability by publishing the source code, and/or providing screenshots/screen-capture video, and/or providing flowcharts of the algorithms used

Ensure replicability by publishing the source code, and/or providing screenshots/screen-capture video, and/or providing flowcharts of the algorithms used. Replicability (i.e., other researchers should in principle be able to replicate the study) is a hallmark of scientific reporting.

	1	2	3	4	5	
subitem not at all important	◉	○	○	○	○	essential

Does your paper address subitem 5-v?

Copy and paste relevant sections from the manuscript (include quotes in quotation marks "like this" to indicate direct quotes from your manuscript), or elaborate on this item by providing additional information not in the ms, or briefly explain why the item is not applicable/relevant for your study

This is a pilot RCT, and this is not relevant.

5-vi) Digital preservation

Digital preservation: Provide the URL of the application, but as the intervention is likely to change or disappear over the course of the years; also make sure the intervention is archived (Internet Archive, webcitation.org, and/or publishing the source code or screenshots/videos alongside the article). As pages behind login screens cannot be archived, consider creating demo pages which are accessible without login.

	1	2	3	4	5	
subitem not at all important	◉	○	○	○	○	essential

Does your paper address subitem 5-vi?

Copy and paste relevant sections from the manuscript (include quotes in quotation marks "like this" to indicate direct quotes from your manuscript), or elaborate on this item by providing additional information not in the ms, or briefly explain why the item is not applicable/relevant for your study

No, we did not.

5-vii) Access

Access: Describe how participants accessed the application, in what setting/context, if they had to pay (or were paid) or not, whether they had to be a member of specific group. If known, describe how participants obtained "access to the platform and Internet" [1]. To ensure access for editors/reviewers/readers, consider to provide a "backdoor" login account or demo mode for reviewers/readers to explore the application (also important for archiving purposes, see vi).

	1	2	3	4	5	
subitem not at all important	○	○	○	○	◉	essential

Does your paper address subitem 5-vii? *

Copy and paste relevant sections from the manuscript (include quotes in quotation marks "like this" to indicate direct quotes from your manuscript), or elaborate on this item by providing additional information not in the ms, or briefly explain why the item is not applicable/relevant for your study

Yes, all access and service is provided free of charge to the users.
"Intervention group participants received a free download and use of a food coaching smartphone app (Glycoleap, Holmusk, Singapore) up to 8 weeks during the RCT."

5-viii) Mode of delivery, features/functionalities/components of the intervention and comparator, and the theoretical framework

Describe mode of delivery, features/functionalities/components of the intervention and comparator, and the theoretical framework [6] used to design them (instructional strategy [1], behaviour change techniques, persuasive features, etc., see e.g., [7, 8] for terminology). This includes an in-depth description of the content (including where it is coming from and who developed it) [1]," whether [and how] it is tailored to individual circumstances and allows users to track their progress and receive feedback" [6]. This also includes a description of communication delivery channels and – if computer-mediated communication is a component – whether communication was synchronous or asynchronous [6]. It also includes information on presentation strategies [1], including page design principles, average amount of text on pages, presence of hyperlinks to other resources, etc. [1].

	1	2	3	4	5	
subitem not at all important	○	○	○	○	◉	essential

Does your paper address subitem 5-viii? *

Copy and paste relevant sections from the manuscript (include quotes in quotation marks "like this" to indicate direct quotes from your manuscript), or elaborate on this item by providing additional information not in the ms, or briefly explain why the item is not applicable/relevant for your study

Yes, it has been mentioned.
"2. Food coaching smartphone app—Intervention group participants received a free download and use of a food coaching smartphone app (Glycoleap, Holmusk, Singapore) up to 8 weeks during the RCT. This food coaching smartphone app was developed by Holmusk, and aimed to improve care and outcomes for people with diabetes. Pregnant women using this app were able to upload food images (e.g., a picture of a meal, a drink, or a dessert) and received real-time and detailed food coaching comments and guidance provided by professional dietitians during the day (8am-8pm). According to the local pregnancy guideline [19], food dietitians rated the food image from 1 to 5 (1 as the least recommended score while 5 as the most recommended scored) and gave feedback in terms of degree and balance of the food items and composition (Supplementary Figure 1)."

5-ix) Describe use parameters

Describe use parameters (e.g., intended “doses” and optimal timing for use). Clarify what instructions or recommendations were given to the user, e.g., regarding timing, frequency, heaviness of use, if any, or was the intervention used ad libitum.

	1	2	3	4	5	
subitem not at all important	(●)	( )	( )	( )	( )	essential

Does your paper address subitem 5-ix?

Copy and paste relevant sections from the manuscript (include quotes in quotation marks "like this" to indicate direct quotes from your manuscript), or elaborate on this item by providing additional information not in the ms, or briefly explain why the item is not applicable/relevant for your study

This is mentioned in the survey form filled up by the smartphone app users. But the doses or optimal timing for use is not applicable or relevant in our study.

5-x) Clarify the level of human involvement

Clarify the level of human involvement (care providers or health professionals, also technical assistance) in the e-intervention or as co-intervention (detail number and expertise of professionals involved, if any, as well as “type of assistance offered, the timing and frequency of the support, how it is initiated, and the medium by which the assistance is delivered”. It may be necessary to distinguish between the level of human involvement required for the trial, and the level of human involvement required for a routine application outside of a RCT setting (discuss under item 21 – generalizability).

	1	2	3	4	5	
subitem not at all important	○	○	○	○	◉	essential

Does your paper address subitem 5-x?

Copy and paste relevant sections from the manuscript (include quotes in quotation marks "like this" to indicate direct quotes from your manuscript), or elaborate on this item by providing additional information not in the ms, or briefly explain why the item is not applicable/relevant for your study

Yes, it has been mentioned.
"This food coaching smartphone app was developed by Holmusk, and aimed to improve care and outcomes for people with diabetes. Pregnant women using this app were able to upload food images (e.g., a picture of a meal, a drink, or a dessert) and received real-time and detailed food coaching comments and guidance provided by professional dietitians during the day (8am-8pm). According to the local pregnancy guideline [19], food dietitians rated the food image from 1 to 5 (1 as the least recommended score while 5 as the most recommended scored) and gave feedback in terms of degree and balance of the food items and composition (Supplementary Figure 1)."

5-xi) Report any prompts/reminders used

Report any prompts/reminders used: Clarify if there were prompts (letters, emails, phone calls, SMS) to use the application, what triggered them, frequency etc. It may be necessary to distinguish between the level of prompts/reminders required for the trial, and the level of prompts/reminders for a routine application outside of a RCT setting (discuss under item 21 – generalizability).

	1	2	3	4	5	
subitem not at all important	◉	○	○	○	○	essential

Does your paper address subitem 5-xi? *

Copy and paste relevant sections from the manuscript (include quotes in quotation marks "like this" to indicate direct quotes from your manuscript), or elaborate on this item by providing additional information not in the ms, or briefly explain why the item is not applicable/relevant for your study

There will be notification prompting in users' cellphone (function embedded in the smartphone app), yet we did not report or remind the user of using the smartphone app frequently. We provided a hotline for problems encountered. Yet the feasibility and the acceptance of delivering such service will be assessed without any additional intervention or reinforcement by the medical staff.

5-xii) Describe any co-interventions (incl. training/support)

Describe any co-interventions (incl. training/support): Clearly state any interventions that are provided in addition to the targeted eHealth intervention, as ehealth intervention may not be designed as stand-alone intervention. This includes training sessions and support [1]. It may be necessary to distinguish between the level of training required for the trial, and the level of training for a routine application outside of a RCT setting (discuss under item 21 – generalizability.

	1	2	3	4	5	
subitem not at all important	(●)	( )	( )	( )	( )	essential

Does your paper address subitem 5-xii? *

Copy and paste relevant sections from the manuscript (include quotes in quotation marks "like this" to indicate direct quotes from your manuscript), or elaborate on this item by providing additional information not in the ms, or briefly explain why the item is not applicable/relevant for your study

There is no co-intervention.

6a) Completely defined pre-specified primary and secondary outcome measures, including how and when they were assessed

Does your paper address CONSORT subitem 6a? *

Copy and paste relevant sections from the manuscript (include quotes in quotation marks "like this" to indicate direct quotes from your manuscript), or elaborate on this item by providing additional information not in the ms, or briefly explain why the item is not applicable/relevant for your study

Yes.
"We retrieved subjects' weight and height (SECA, Vogel and Halke, Germany) and blood pressure (Omron HEM 705 LP, Omron Healthcare Inc, USA) at three time points (baseline and two follow-up visits) from medical records. Body mass index is calculated as the ratio of weight (kg) over the square of height (m). We defined and calculated the proportion with optimal GWG based on the Institute of Medicine (IOM) guidelines—for overweight women: 0.23-0.33 kg/week; and for obese women: 0.17-0.27 kg/week, during the 2nd trimester respectively [20,21].

We assisted all pregnant subjects in quantifying their food and beverage intake. Visual aids such as standardized household measuring utensils and food pictures of various portion sizes were presented. After familiarizing with the food pictures, trained interviewers led the subjects through with a 3-step multiple-pass assessment: 1) the itemized dietary intake list, which is a listing by the subjects of foods and beverages consumed; 2) a time and occasion at which foods were consumed; 3) the detailed cycle, which elicits descriptions of food items and ways of cooking, together with amounts eaten aided by the interactive use of the food pictures and measuring guides. Results from 24-hour food recall and 3-day food diary and those from food frequency questionnaires have been shown to be comparable [22]. Such methods of assessing dietary data have been widely published and accepted [23-25]. We used the same form for collecting 24-hour food recall and 3-day food diary (Supplementary Table 1). We did a 24-hour food recall at recruitment, and required all pregnant women in both groups to follow the example and complete the 3-day food diary at home, inclusive of 2 week days and 1 weekend. We used weekend diary to validate the dietary data obtained from the weekday diary. We collected three copies of 3-day food diary forms at the 4-week (22-24 weeks gestation) and 8-week (26-28 weeks gestation) follow-up visits.

We analyzed the dietary records using HPB online nutrient analysis software [26] which was derived on locally available foods [19,22,27]. Total energy and macronutrient intakes were listed in kilocalories, grams and percentages in the data summary form."

6a-i) Online questionnaires: describe if they were validated for online use and apply CHERRIES items to describe how the questionnaires were designed/deployed

If outcomes were obtained through online questionnaires, describe if they were validated for online use and apply CHERRIES items to describe how the questionnaires were designed/deployed [9].

	1	2	3	4	5	
subitem not at all important	○	◉	○	○	○	essential

Does your paper address subitem 6a-i?

Copy and paste relevant sections from manuscript text

It is designed to assess the feasibility and acceptance of the smartphone app. However, it is not available online. We described and also attached the questionnaires in the supplementary materials.

"We encouraged all pregnant women in intervention group to fill up the user evaluation form at the 8-week follow-up visit (Supplementary Figure 2), to assess the feasibility and collect participants' feedback."

6a-ii) Describe whether and how "use" (including intensity of use/dosage) was defined/measured/monitored

Describe whether and how "use" (including intensity of use/dosage) was defined/measured/monitored (logins, logfile analysis, etc.). Use/adoption metrics are important process outcomes that should be reported in any ehealth trial.

	1	2	3	4	5	
subitem not at all important	○	○	◉	○	○	essential

Does your paper address subitem 6a-ii?

Copy and paste relevant sections from manuscript text

"Based on the log-in records, the uptake of this smartphone app among our intervention subjects was up to 90% at the beginning and 70% at the end of the study, respectively. Based on 12 returned user evaluation forms, 11 subjects (91.7%) were satisfied and would recommend the smartphone app to family and friends. Among 6 mothers who were highly satisfied with the smartphone App, they indicated that such food recommendation had “changed their choices of food, portion of food, and more inclined to take fruits, vegetables and water instead of meat or soft drink.”

6a-iii) Describe whether, how, and when qualitative feedback from participants was obtained

Describe whether, how, and when qualitative feedback from participants was obtained (e.g., through emails, feedback forms, interviews, focus groups).

	1	2	3	4	5	
subitem not at all important	○	○	◉	○	○	essential

Does your paper address subitem 6a-iii?

Copy and paste relevant sections from manuscript text

"Based on the log-in records, the uptake of this smartphone app among our intervention subjects was up to 90% at the beginning and 70% at the end of the study, respectively. Based on 12 returned user evaluation forms, 11 subjects (91.7%) were satisfied and would recommend the smartphone app to family and friends. Among 6 mothers who were highly satisfied with the smartphone App, they indicated that such food recommendation had “changed their choices of food, portion of food, and more inclined to take fruits, vegetables and water instead of meat or soft drink.”

6b) Any changes to trial outcomes after the trial commenced, with reasons

Does your paper address CONSORT subitem 6b? *

Copy and paste relevant sections from the manuscript (include quotes in quotation marks "like this" to indicate direct quotes from your manuscript), or elaborate on this item by providing additional information not in the ms, or briefly explain why the item is not applicable/relevant for your study

No, there is no changes made to trial outcomes after the trial commenced.

7a) How sample size was determined

NPT: When applicable, details of whether and how the clustering by care provides or centers was addressed

7a-i) Describe whether and how expected attrition was taken into account when calculating the sample size

Describe whether and how expected attrition was taken into account when calculating the sample size.

	1	2	3	4	5	
subitem not at all important	(•)	( )	( )	( )	( )	essential

Does your paper address subitem 7a-i?

Copy and paste relevant sections from manuscript title (include quotes in quotation marks "like this" to indicate direct quotes from your manuscript), or elaborate on this item by providing additional information not in the ms, or briefly explain why the item is not applicable/relevant for your study

This is a pilot, therefore, the sample size was set at 15 intervention cases and 15 controls to preliminarily assess feasibility and acceptance, and to further determine the sample size calculation in a bigger RCT if the results are promising.

7b) When applicable, explanation of any interim analyses and stopping guidelines

Does your paper address CONSORT subitem 7b? *

Copy and paste relevant sections from the manuscript (include quotes in quotation marks "like this" to indicate direct quotes from your manuscript), or elaborate on this item by providing additional information not in the ms, or briefly explain why the item is not applicable/relevant for your study

No, it is not mentioned. It is not applicable.

8a) Method used to generate the random allocation sequence

NPT: When applicable, how care providers were allocated to each trial group

Does your paper address CONSORT subitem 8a? *

Copy and paste relevant sections from the manuscript (include quotes in quotation marks "like this" to indicate direct quotes from your manuscript), or elaborate on this item by providing additional information not in the ms, or briefly explain why the item is not applicable/relevant for your study

Yes, we used envelop randomization.
"We randomly assigned pregnant women to intervention group (using food coaching smartphone app) or to control group (receiving standard pregnancy dietary orientation) in a 1:1 allocation ratio, using the envelopment randomization method. Pregnant women, investigators and study staff were not masked to group allocation (Figure 1)."

8b) Type of randomisation; details of any restriction (such as blocking and block size)

Does your paper address CONSORT subitem 8b? *

Copy and paste relevant sections from the manuscript (include quotes in quotation marks "like this" to indicate direct quotes from your manuscript), or elaborate on this item by providing additional information not in the ms, or briefly explain why the item is not applicable/relevant for your study

We did not detail any restriction for the type of randomisation since this is a pilot study.

9) Mechanism used to implement the random allocation sequence (such as sequentially numbered containers), describing any steps taken to conceal the sequence until interventions were assigned

Does your paper address CONSORT subitem 9? *

Copy and paste relevant sections from the manuscript (include quotes in quotation marks "like this" to indicate direct quotes from your manuscript), or elaborate on this item by providing additional information not in the ms, or briefly explain why the item is not applicable/relevant for your study

No, this is a pilot study. Detailed mechanism used to implement the random allocation sequence is not applicable.

10) Who generated the random allocation sequence, who enrolled participants, and who assigned participants to interventions

Does your paper address CONSORT subitem 10? *

Copy and paste relevant sections from the manuscript (include quotes in quotation marks "like this" to indicate direct quotes from your manuscript), or elaborate on this item by providing additional information not in the ms, or briefly explain why the item is not applicable/relevant for your study

Yes, the medical research coordinator.
"Research coordinators randomly assigned pregnant women to intervention group (using food coaching smartphone app) or to control group (receiving standard pregnancy dietary orientation) in a 1:1 allocation ratio, using the envelopment randomization method. Pregnant women, investigators and study staff were not masked to group allocation (Figure 1). "

11a) If done, who was blinded after assignment to interventions (for example, participants, care providers, those assessing outcomes) and how

NPT: Whether or not administering co-interventions were blinded to group assignment

11a-i) Specify who was blinded, and who wasn’t

Specify who was blinded, and who wasn’t. Usually, in web-based trials it is not possible to blind the participants [1, 3] (this should be clearly acknowledged), but it may be possible to blind outcome assessors, those doing data analysis or those administering co-interventions (if any).

	1	2	3	4	5	
subitem not at all important	○	○	○	○	●	essential

Does your paper address subitem 11a-i? *

Copy and paste relevant sections from the manuscript (include quotes in quotation marks "like this" to indicate direct quotes from your manuscript), or elaborate on this item by providing additional information not in the ms, or briefly explain why the item is not applicable/relevant for your study

This is not blinded.
"Pregnant women, investigators and study staff were not masked to group allocation."

11a-ii) Discuss e.g., whether participants knew which intervention was the “intervention of interest” and which one was the “comparator”

Informed consent procedures (4a-ii) can create biases and certain expectations - discuss e.g., whether participants knew which intervention was the “intervention of interest” and which one was the “comparator”.

	1	2	3	4	5	
subitem not at all important	○	○	○	○	◉	essential

Does your paper address subitem 11a-ii?

Copy and paste relevant sections from the manuscript (include quotes in quotation marks "like this" to indicate direct quotes from your manuscript), or elaborate on this item by providing additional information not in the ms, or briefly explain why the item is not applicable/relevant for your study

"Pregnant women, investigators and study staff were not masked to group allocation."

11b) If relevant, description of the similarity of interventions

(this item is usually not relevant for ehealth trials as it refers to similarity of a placebo or sham intervention to a active medication/intervention)

Does your paper address CONSORT subitem 11b? *

Copy and paste relevant sections from the manuscript (include quotes in quotation marks "like this" to indicate direct quotes from your manuscript), or elaborate on this item by providing additional information not in the ms, or briefly explain why the item is not applicable/relevant for your study

No, this is not relevant.

12a) Statistical methods used to compare groups for primary and secondary outcomes

NPT: When applicable, details of whether and how the clustering by care providers or centers was addressed

Does your paper address CONSORT subitem 12a? *

Copy and paste relevant sections from the manuscript (include quotes in quotation marks "like this" to indicate direct quotes from your manuscript), or elaborate on this item by providing additional information not in the ms, or briefly explain why the item is not applicable/relevant for your study

"We reported mean and standard deviation (SD) for continuous variables, and counts and percentages for categorical variables. We applied Fisher's exact test and Student's t-tests for categorical and continuous variables to compare characteristics between intervention and control groups.

We used linear regression to examine the effect of food coaching smartphone app on weight gain control and macronutrient intake between two groups. We performed statistical analysis using STATA (Version 14.0, STATA Corp, Texas, US). We set a two-tailed p-value for significance at .05 and provided the 95% confidence intervals (CI) for all estimates."

12a-i) Imputation techniques to deal with attrition / missing values

Imputation techniques to deal with attrition / missing values: Not all participants will use the intervention/comparator as intended and attrition is typically high in ehealth trials. Specify how participants who did not use the application or dropped out from the trial were treated in the statistical analysis (a complete case analysis is strongly discouraged, and simple imputation techniques such as LOCF may also be problematic [4]).

	1	2	3	4	5	
subitem not at all important	(●)	( )	( )	( )	( )	essential

Does your paper address subitem 12a-i? *

Copy and paste relevant sections from the manuscript (include quotes in quotation marks "like this" to indicate direct quotes from your manuscript), or elaborate on this item by providing additional information not in the ms, or briefly explain why the item is not applicable/relevant for your study

Imputation techniques are not required for such a small sample.

12b) Methods for additional analyses, such as subgroup analyses and adjusted analyses

Does your paper address CONSORT subitem 12b? *

Copy and paste relevant sections from the manuscript (include quotes in quotation marks "like this" to indicate direct quotes from your manuscript), or elaborate on this item by providing additional information not in the ms, or briefly explain why the item is not applicable/relevant for your study

No, this study only contains a small sample, therefore, subgroup analyses are not applicable.

X26) REB/IRB Approval and Ethical Considerations [recommended as subheading under "Methods"] (not a CONSORT item)

X26-i) Comment on ethics committee approval

	1	2	3	4	5	
subitem not at all important	○	○	○	○	◉	essential

Does your paper address subitem X26-i?

Copy and paste relevant sections from the manuscript (include quotes in quotation marks "like this" to indicate direct quotes from your manuscript), or elaborate on this item by providing additional information not in the ms, or briefly explain why the item is not applicable/relevant for your study

"We conducted the study according to the tenets of the Declaration of Helsinki, and obtained approval by the SingHealth Centralized Institutional Review Board and the National Health Group’s Domain Specific Review Board. We obtained written informed consents from all pregnant women at baseline recruitment."

x26-ii) Outline informed consent procedures

Outline informed consent procedures e.g., if consent was obtained offline or online (how? Checkbox, etc.?), and what information was provided (see 4a-ii). See [6] for some items to be included in informed consent documents.

	1	2	3	4	5	
subitem not at all important	○	○	○	○	◉	essential

Does your paper address subitem X26-ii?

Copy and paste relevant sections from the manuscript (include quotes in quotation marks "like this" to indicate direct quotes from your manuscript), or elaborate on this item by providing additional information not in the ms, or briefly explain why the item is not applicable/relevant for your study

"We conducted the study according to the tenets of the Declaration of Helsinki, and obtained approval by the SingHealth Centralized Institutional Review Board and the National Health Group's Domain Specific Review Board. We obtained written informed consents from all pregnant women at baseline recruitment."

X26-iii) Safety and security procedures

Safety and security procedures, incl. privacy considerations, and any steps taken to reduce the likelihood or detection of harm (e.g., education and training, availability of a hotline)

	1	2	3	4	5	
subitem not at all important	◉	○	○	○	○	essential

Does your paper address subitem X26-iii?

Copy and paste relevant sections from the manuscript (include quotes in quotation marks "like this" to indicate direct quotes from your manuscript), or elaborate on this item by providing additional information not in the ms, or briefly explain why the item is not applicable/relevant for your study

We have indicated such in the IRB consent form, yet it is not relevant to the paper itself.

RESULTS

13a) For each group, the numbers of participants who were randomly assigned, received intended treatment, and were analysed for the primary outcome

NPT: The number of care providers or centers performing the intervention in each group and the number of patients treated by each care provider in each center

Does your paper address CONSORT subitem 13a? *

Copy and paste relevant sections from the manuscript (include quotes in quotation marks "like this" to indicate direct quotes from your manuscript), or elaborate on this item by providing additional information not in the ms, or briefly explain why the item is not applicable/relevant for your study

"Even though we did envelop randomization for the group allocation, the intervention group had slightly higher BMI (34.2 vs. 31.3 kg/m2, p=0.04) and diastolic blood pressure at baseline (69.4 vs. 64.1 mm Hg, p=0.03), compared with control group. We observed no differences in age, ethnicity, smoking history, parity, maternal and paternal college degree, pre-pregnancy BMI and past pregnancy complications between intervention and control groups (Table 1)."

13b) For each group, losses and exclusions after randomisation, together with reasons

Does your paper address CONSORT subitem 13b? (NOTE: Preferably, this is shown in a CONSORT flow diagram) *

Copy and paste relevant sections from the manuscript (include quotes in quotation marks "like this" to indicate direct quotes from your manuscript), or elaborate on this item by providing additional information not in the ms, or briefly explain why the item is not applicable/relevant for your study

Yes, shown in Figure 1.
"A total of 30 pregnant women were recruited at baseline. Both intervention (n=15) and control (n=15) groups received standard pregnancy dietary orientation, while the intervention group additionally used food coaching smartphone app. In the 4-week's follow-up, three women dropped out from the intervention group and in the 8-week's follow-up, one woman gave birth prematurely in the control group. A total of 12 women in the intervention group and 14 women in the control group completed the RCT (Figure 1)."

13b-i) Attrition diagram

Strongly recommended: An attrition diagram (e.g., proportion of participants still logging in or using the intervention/comparator in each group plotted over time, similar to a survival curve) or other figures or tables demonstrating usage/dose/engagement.

	1	2	3	4	5	
subitem not at all important	◉	○	○	○	○	essential

Does your paper address subitem 13b-i?

Copy and paste relevant sections from the manuscript or cite the figure number if applicable (include quotes in quotation marks "like this" to indicate direct quotes from your manuscript), or elaborate on this item by providing additional information not in the ms, or briefly explain why the item is not applicable/relevant for your study

We did not address this since this is not the primary or secondary outcome of this study.

14a) Dates defining the periods of recruitment and follow-up

Does your paper address CONSORT subitem 14a? *

Copy and paste relevant sections from the manuscript (include quotes in quotation marks "like this" to indicate direct quotes from your manuscript), or elaborate on this item by providing additional information not in the ms, or briefly explain why the item is not applicable/relevant for your study

Yes, we provided the dates and the duration of the study.
"We conducted a prospective, two-arm (n=30) and unblinded randomized controlled trial (RCT) in a subsidized clinic within a tertiary government hospital in Singapore (KK Women's and Children's Hospital; KKH), between March and July, 2018."
"A total of 30 pregnant women were recruited at baseline. Both intervention (n=15) and control (n=15) groups received standard pregnancy dietary orientation, while the intervention group additionally used food coaching smartphone app. In the 4-week's follow-up, three women dropped out from the intervention group and in the 8-week's follow-up, one woman gave birth prematurely in the control group. A total of 12 women in the intervention group and 14 women in the control group completed the RCT (Figure 1)."

14a-i) Indicate if critical “secular events” fell into the study period

Indicate if critical “secular events” fell into the study period, e.g., significant changes in Internet resources available or “changes in computer hardware or Internet delivery resources”

	1	2	3	4	5	
subitem not at all important	(•)	( )	( )	( )	( )	essential

Does your paper address subitem 14a-i?

Copy and paste relevant sections from the manuscript (include quotes in quotation marks "like this" to indicate direct quotes from your manuscript), or elaborate on this item by providing additional information not in the ms, or briefly explain why the item is not applicable/relevant for your study

We did not include this into the study since this is not the primary or secondary outcome of the study.

14b) Why the trial ended or was stopped (early)

Does your paper address CONSORT subitem 14b? *

Copy and paste relevant sections from the manuscript (include quotes in quotation marks "like this" to indicate direct quotes from your manuscript), or elaborate on this item by providing additional information not in the ms, or briefly explain why the item is not applicable/relevant for your study

This is not a stopped or early ended trial. So it is not applicable.

15) A table showing baseline demographic and clinical characteristics for each group

NPT: When applicable, a description of care providers (case volume, qualification, expertise, etc.) and centers (volume) in each group

Does your paper address CONSORT subitem 15? *

Copy and paste relevant sections from the manuscript (include quotes in quotation marks "like this" to indicate direct quotes from your manuscript), or elaborate on this item by providing additional information not in the ms, or briefly explain why the item is not applicable/relevant for your study

Yes, please kindly refer to Table 1 and Table 2.
"Even though we did envelop randomization for the group allocation, the intervention group had slightly higher BMI (34.2 vs. 31.3 kg/m2, p=0.04) and diastolic blood pressure at baseline (69.4 vs. 64.1 mm Hg, p=0.03), compared with control group. We observed no differences in age, ethnicity, smoking history, parity, maternal and paternal college degree, pre-pregnancy BMI and past pregnancy complications between intervention and control groups (Table 1).

Table 2 shows more cases obtaining the IOM recommendation on optimal GWG per week in the intervention group than the control group at the 4-week (58.3 % vs. 53.3%, p=0.67) and 8-week follow-up visits (58.3% vs. 35.7%, p=0.43), respectively. Furthermore, the intervention group consistently had higher BMI at the 4-week (36.4 vs. 31.9 kg/ m2, p<0.01) and 8-week follow-up visits (36.0 vs. 32.4 kg/ m2, p=0.02), compared with control group. However, there were no significant differences in BP, GWG since baseline, total energy intake and all macronutrients intakes between the two groups at either follow-up visits (Table 2)."

15-i) Report demographics associated with digital divide issues

In ehealth trials it is particularly important to report demographics associated with digital divide issues, such as age, education, gender, social-economic status, computer/Internet/ehealth literacy of the participants, if known.

	1	2	3	4	5	
subitem not at all important	○	○	○	○	●	essential

Does your paper address subitem 15-i? *

Copy and paste relevant sections from the manuscript (include quotes in quotation marks "like this" to indicate direct quotes from your manuscript), or elaborate on this item by providing additional information not in the ms, or briefly explain why the item is not applicable/relevant for your study

"Even though we did envelop randomization for the group allocation, the intervention group had slightly higher BMI (34.2 vs. 31.3 kg/m2, p=0.04) and diastolic blood pressure at baseline (69.4 vs. 64.1 mm Hg, p=0.03), compared with control group. We observed no differences in age, ethnicity, smoking history, parity, maternal and paternal college degree, pre-pregnancy BMI and past pregnancy complications between intervention and control groups (Table 1). "

16) For each group, number of participants (denominator) included in each analysis and whether the analysis was by original assigned groups

16-i) Report multiple "denominators" and provide definitions

Report multiple "denominators" and provide definitions: Report N's (and effect sizes) "across a range of study participation [and use] thresholds" [1], e.g., N exposed, N consented, N used more than x times, N used more than y weeks, N participants "used" the intervention/comparator at specific pre-defined time points of interest (in absolute and relative numbers per group). Always clearly define "use" of the intervention.

	1	2	3	4	5	
subitem not at all important	○	○	○	○	◉	essential

Does your paper address subitem 16-i? *

Copy and paste relevant sections from the manuscript (include quotes in quotation marks "like this" to indicate direct quotes from your manuscript), or elaborate on this item by providing additional information not in the ms, or briefly explain why the item is not applicable/relevant for your study

Yes, the denominator is the baseline readings.
"We found that women in the intervention group tended to have less weight gain than the control group at the 4-week (mean difference -0.15 kg, 9%% CI: -1.51, 1.21; p=0.83) and 8-week follow-up (-0.08 kg, 95% CI: -1.80, 1.63; p=0.92) (Table 3). Also, women in the intervention group tended to consume less cholesterol than the control group at the 4-week (mean difference -64.87 mg, 95% CI: -146.04, 16.31; p=0.11) and 8-week follow-up (-31.73 mg, 95% CI: -102.91, 39.45; p=0.37) (Table 3). These associations however, were not statistically significant in our limited sample size. Furthermore, based on the nature of the RCT study design, we did not adjust for any variable in our linear regression, or performed sensitivity analysis."

16-ii) Primary analysis should be intent-to-treat

Primary analysis should be intent-to-treat, secondary analyses could include comparing only “users”, with the appropriate caveats that this is no longer a randomized sample (see 18-i).

	1	2	3	4	5	
subitem not at all important	○	○	○	○	●	essential

Does your paper address subitem 16-ii?

Copy and paste relevant sections from the manuscript (include quotes in quotation marks "like this" to indicate direct quotes from your manuscript), or elaborate on this item by providing additional information not in the ms, or briefly explain why the item is not applicable/relevant for your study

"We found that women in the intervention group tended to have less weight gain than the control group at the 4-week (mean difference -0.15 kg, 9%% CI: -1.51, 1.21; p=0.83) and 8-week follow-up (-0.08 kg, 95% CI: -1.80, 1.63; p=0.92) (Table 3). Also, women in the intervention group tended to consume less cholesterol than the control group at the 4-week (mean difference -64.87 mg, 95% CI: -146.04, 16.31; p=0.11) and 8-week follow-up (-31.73 mg, 95% CI: -102.91, 39.45; p=0.37) (Table 3). These associations however, were not statistically significant in our limited sample size. Furthermore, based on the nature of the RCT study design, we did not adjust for any variable in our linear regression, or performed sensitivity analysis. "

17a) For each primary and secondary outcome, results for each group, and the estimated effect size and its precision (such as 95% confidence interval)

Does your paper address CONSORT subitem 17a? *

Copy and paste relevant sections from the manuscript (include quotes in quotation marks "like this" to indicate direct quotes from your manuscript), or elaborate on this item by providing additional information not in the ms, or briefly explain why the item is not applicable/relevant for your study

Yes, 95% CI and p value are both provided.
"We found that women in the intervention group tended to have less weight gain than the control group at the 4-week (mean difference -0.15 kg, 9%% CI: -1.51, 1.21; p=0.83) and 8-week follow-up (-0.08 kg, 95% CI: -1.80, 1.63; p=0.92) (Table 3). Also, women in the intervention group tended to consume less cholesterol than the control group at the 4-week (mean difference -64.87 mg, 95% CI: -146.04, 16.31; p=0.11) and 8-week follow-up (-31.73 mg, 95% CI: -102.91, 39.45; p=0.37) (Table 3). These associations however, were not statistically significant in our limited sample size. Furthermore, based on the nature of the RCT study design, we did not adjust for any variable in our linear regression, or performed sensitivity analysis."

17a-i) Presentation of process outcomes such as metrics of use and intensity of use

In addition to primary/secondary (clinical) outcomes, the presentation of process outcomes such as metrics of use and intensity of use (dose, exposure) and their operational definitions is critical. This does not only refer to metrics of attrition (13-b) (often a binary variable), but also to more continuous exposure metrics such as “average session length”. These must be accompanied by a technical description how a metric like a “session” is defined (e.g., timeout after idle time) [1] (report under item 6a).

	1	2	3	4	5	
subitem not at all important	◉	○	○	○	○	essential

Does your paper address subitem 17a-i?

Copy and paste relevant sections from the manuscript (include quotes in quotation marks "like this" to indicate direct quotes from your manuscript), or elaborate on this item by providing additional information not in the ms, or briefly explain why the item is not applicable/relevant for your study

We did not go into such detailed analyses, due to the fact that we only have a small sample.

17b) For binary outcomes, presentation of both absolute and relative effect sizes is recommended

Does your paper address CONSORT subitem 17b? *

Copy and paste relevant sections from the manuscript (include quotes in quotation marks "like this" to indicate direct quotes from your manuscript), or elaborate on this item by providing additional information not in the ms, or briefly explain why the item is not applicable/relevant for your study

We do not have binary outcomes.

18) Results of any other analyses performed, including subgroup analyses and adjusted analyses, distinguishing pre-specified from exploratory

Does your paper address CONSORT subitem 18? *

Copy and paste relevant sections from the manuscript (include quotes in quotation marks "like this" to indicate direct quotes from your manuscript), or elaborate on this item by providing additional information not in the ms, or briefly explain why the item is not applicable/relevant for your study

We did not perform subgroup analyses.

18-i) Subgroup analysis of comparing only users

A subgroup analysis of comparing only users is not uncommon in ehealth trials, but if done, it must be stressed that this is a self-selected sample and no longer an unbiased sample from a randomized trial (see 16-iii).

	1	2	3	4	5	
subitem not at all important	◉	○	○	○	○	essential

Does your paper address subitem 18-i?

Copy and paste relevant sections from the manuscript (include quotes in quotation marks "like this" to indicate direct quotes from your manuscript), or elaborate on this item by providing additional information not in the ms, or briefly explain why the item is not applicable/relevant for your study

Small sample size restricted such further analysis.

19) All important harms or unintended effects in each group

(for specific guidance see CONSORT for harms)

Does your paper address CONSORT subitem 19? *

Copy and paste relevant sections from the manuscript (include quotes in quotation marks "like this" to indicate direct quotes from your manuscript), or elaborate on this item by providing additional information not in the ms, or briefly explain why the item is not applicable/relevant for your study

No, it is not applicable since the smartphone app will not harm or affect the users at all.

19-i) Include privacy breaches, technical problems

Include privacy breaches, technical problems. This does not only include physical “harm” to participants, but also incidents such as perceived or real privacy breaches [1], technical problems, and other unexpected/unintended incidents. “Unintended effects” also includes unintended positive effects [2].

	1	2	3	4	5	
subitem not at all important	(●)	( )	( )	( )	( )	essential

Does your paper address subitem 19-i?

Copy and paste relevant sections from the manuscript (include quotes in quotation marks "like this" to indicate direct quotes from your manuscript), or elaborate on this item by providing additional information not in the ms, or briefly explain why the item is not applicable/relevant for your study

All patients registered with a study ID. They only provided their email for registration. The company or the backend dietitian or nutritionist would not be able to know the privacy of study subjects. So this is not applicable.

19-ii) Include qualitative feedback from participants or observations from staff/researchers

Include qualitative feedback from participants or observations from staff/researchers, if available, on strengths and shortcomings of the application, especially if they point to unintended/unexpected effects or uses. This includes (if available) reasons for why people did or did not use the application as intended by the developers.

	1	2	3	4	5	
subitem not at all important	○	○	○	○	◉	essential

Does your paper address subitem 19-ii?

Copy and paste relevant sections from the manuscript (include quotes in quotation marks "like this" to indicate direct quotes from your manuscript), or elaborate on this item by providing additional information not in the ms, or briefly explain why the item is not applicable/relevant for your study

We provided the qualitative feedback from participants.
"Based on the log-in records, the uptake of this smartphone app among our intervention subjects was up to 90% at the beginning and 70% at the end of the study, respectively. Based on 12 returned user evaluation forms, 11 subjects (91.7%) were satisfied and would recommend the smartphone app to family and friends. Among 6 mothers who were highly satisfied with the smartphone App, they indicated that such food recommendation had “changed their choices of food, portion of food, and more inclined to take fruits, vegetables and water instead of meat or soft drink."

DISCUSSION

22) Interpretation consistent with results, balancing benefits and harms, and considering other relevant evidence

NPT: In addition, take into account the choice of the comparator, lack of or partial blinding, and unequal expertise of care providers or centers in each group

22-i) Restate study questions and summarize the answers suggested by the data, starting with primary outcomes and process outcomes (use)

Restate study questions and summarize the answers suggested by the data, starting with primary outcomes and process outcomes (use).

	1	2	3	4	5	
subitem not at all important	○	○	○	○	◉	essential

Does your paper address subitem 22-i? *

Copy and paste relevant sections from the manuscript (include quotes in quotation marks "like this" to indicate direct quotes from your manuscript), or elaborate on this item by providing additional information not in the ms, or briefly explain why the item is not applicable/relevant for your study

In this study, we adopted the technology of delivering dietary recommendation via an e-interface approach—smartphone app—to guide overweight and obese pregnant women to eat heathier in order to obtain optimal weight gain, in a small sample RCT. We not only received high uptake of the smartphone as described above, but also found evidence that pregnant women in intervention group were more inclined to gain less weight and consume less cholesterol, compared with controls. Although our results were not significant (which could be attributed to the small sample size), it provided a proof-of-concept on the feasibility of applying such technology in future RCTs with a larger sample size, an earlier intervention onset, and a longer follow-up for overweight and obese pregnant women.

22-ii) Highlight unanswered new questions, suggest future research

Highlight unanswered new questions, suggest future research.

	1	2	3	4	5	
subitem not at all important	○	○	○	○	◉	essential

Does your paper address subitem 22-ii?

Copy and paste relevant sections from the manuscript (include quotes in quotation marks "like this" to indicate direct quotes from your manuscript), or elaborate on this item by providing additional information not in the ms, or briefly explain why the item is not applicable/relevant for your study

"While there is no doubt that increasing the intensity of an intervention may improve pregnancy outcomes, implementing such intense regimens and lengthy consultation into clinical practice may, however, cast a huge burden on both health providers and the pregnant women themselves. Third, the evidence surrounding technology-supported interventions as a safe and sustainable tool for lifestyle interventions in pregnancy still remains understudied [32].

Compared with traditional methods (e.g., leaflets and verbal consultation), apps are more flexible, interactive and have more variable modes of communication (text, pictures, sound, interactivity, etc.) Findings from two RCTs in Norway [33] and Ireland [34] suggest that mobile-Health care model is feasible in adult patients and may be an important method to enhance self-management when delivered in combination with health counseling. In addition, the feasibility and efficacy of smartphone app have been proven in weight management among pediatric obese patients [16] and in glucose control in mothers with GDM during pregnancy [17]. Given the high smartphone usage rate (up to 80%) among Singaporeans[35] and high prevalence (up to 30%) of overweight and obesity among pregnant women in tertiary hospital setting [10], applying a mobile app to promote healthy dietary intake is feasible and likely a promising intervention strategy. Overall, during an 8-week's intervention, pregnant subjects utilizing the smartphone app were likely to have optimal gestational weight than controls. Our findings showed feasibility and acceptability of such food coaching smartphone app. For example, 9 out of 12 (75.0%) smartphone app users found the app is very easy to operate. Ten out of 12 (83.3%) users thought the food coaching guidance was acceptably fast, and 11 out 12 users thought the app had somewhat or greatly supervised their own diet. And it is promising to conduct a further investigation on the utility and efficacy on such an app, controlling weight gain and reduce risk of macrosomia and GDM throughout pregnancy. "

20) Trial limitations, addressing sources of potential bias, imprecision, and, if relevant, multiplicity of analyses

20-i) Typical limitations in ehealth trials

Typical limitations in ehealth trials: Participants in ehealth trials are rarely blinded. Ehealth trials often look at a multiplicity of outcomes, increasing risk for a Type I error. Discuss biases due to non-use of the intervention/usability issues, biases through informed consent procedures, unexpected events.

	1	2	3	4	5	
subitem not at all important	○	○	○	○	◉	essential

Does your paper address subitem 20-i? *

Copy and paste relevant sections from the manuscript (include quotes in quotation marks "like this" to indicate direct quotes from your manuscript), or elaborate on this item by providing additional information not in the ms, or briefly explain why the item is not applicable/relevant for your study

"The strength of our study included a prospective RCT study design, trained staff to provide good standard pregnancy dietary recommendation, and standard protocols in anthropometric measures and dietary assessments. However, our study is not without limitations. First, the baseline BMI seemed to be higher in the intervention group, suggesting that the envelop randomization might not have effectively removing the confounding of weight in both groups. Second, the loss of follow-up in both groups might affect our observed effect size. Third, all our linear regressions and most of the comparisons were not significant due to a lack of study power. However, given that the effect estimates fell in the desired direction, it supports the proof-of-concept as the primary focus in this pilot RCT."

21) Generalisability (external validity, applicability) of the trial findings

NPT: External validity of the trial findings according to the intervention, comparators, patients, and care providers or centers involved in the trial

21-i) Generalizability to other populations

Generalizability to other populations: In particular, discuss generalizability to a general Internet population, outside of a RCT setting, and general patient population, including applicability of the study results for other organizations

	1	2	3	4	5	
subitem not at all important	○	◉	○	○	○	essential

Does your paper address subitem 21-i?

Copy and paste relevant sections from the manuscript (include quotes in quotation marks "like this" to indicate direct quotes from your manuscript), or elaborate on this item by providing additional information not in the ms, or briefly explain why the item is not applicable/relevant for your study

This is a pilot, therefore the discussion of generalisability is too early to make. "Moving forward, we will adopt such food coaching smartphone app and test its utility in a larger setting with targeted population, earlier intervention and longer follow-up throughout pregnancy."

21-ii) Discuss if there were elements in the RCT that would be different in a routine application setting

Discuss if there were elements in the RCT that would be different in a routine application setting (e.g., prompts/reminders, more human involvement, training sessions or other co-interventions) and what impact the omission of these elements could have on use, adoption, or outcomes if the intervention is applied outside of a RCT setting.

	1	2	3	4	5	
subitem not at all important	(●)	○	○	○	○	essential

Does your paper address subitem 21-ii?

Copy and paste relevant sections from the manuscript (include quotes in quotation marks "like this" to indicate direct quotes from your manuscript), or elaborate on this item by providing additional information not in the ms, or briefly explain why the item is not applicable/relevant for your study

This is not the focus of the study, therefore, it is not discussed.

OTHER INFORMATION

23) Registration number and name of trial registry

Does your paper address CONSORT subitem 23? *

Copy and paste relevant sections from the manuscript (include quotes in quotation marks "like this" to indicate direct quotes from your manuscript), or elaborate on this item by providing additional information not in the ms, or briefly explain why the item is not applicable/relevant for your study

This is not applicable. Our pilot RCT received the IRB approval from Singapore National Medical Research Centre (NMRC) with CIRB 2017/2132. However, due to the small sample size of the study, and the aim of this study is just to test the feasibility of the smartphone app, registration of the RCT is not applied in our study.

24) Where the full trial protocol can be accessed, if available

Does your paper address CONSORT subitem 24? *

Cite a Multimedia Appendix, other reference, or copy and paste relevant sections from the manuscript (include quotes in quotation marks "like this" to indicate direct quotes from your manuscript), or elaborate on this item by providing additional information not in the ms, or briefly explain why the item is not applicable/relevant for your study

The full trail's proposal has been drafted up based on the pilot data and sent out for submission. Once it is granted, the registration number will be applied.

25) Sources of funding and other support (such as supply of drugs), role of funders

Does your paper address CONSORT subitem 25? *

Copy and paste relevant sections from the manuscript (include quotes in quotation marks "like this" to indicate direct quotes from your manuscript), or elaborate on this item by providing additional information not in the ms, or briefly explain why the item is not applicable/relevant for your study

Yes.
"This study is supported by Singapore National Medical Research Council Centre Grant [NMRC/CG/C008A/2017_KKH]. Dr. Li is funded by Singapore National Medical Research Council Transition Award [NMRC TA/0027/2014] and [NMRC/CG/C008A/2017_KKH]."

X27) Conflicts of Interest (not a CONSORT item)

X27-i) State the relation of the study team towards the system being evaluated

In addition to the usual declaration of interests (financial or otherwise), also state the relation of the study team towards the system being evaluated, i.e., state if the authors/evaluators are distinct from or identical with the developers/sponsors of the intervention.

	1	2	3	4	5	
subitem not at all important	○	○	○	○	◉	essential

Does your paper address subitem X27-i?

Copy and paste relevant sections from the manuscript (include quotes in quotation marks "like this" to indicate direct quotes from your manuscript), or elaborate on this item by providing additional information not in the ms, or briefly explain why the item is not applicable/relevant for your study

"The authors declared no conflict of interest."

About the CONSORT EHEALTH checklist

As a result of using this checklist, did you make changes in your manuscript? *

- ○ yes, major changes
- ◉ yes, minor changes
- ○ no

What were the most important changes you made as a result of using this checklist?

Just re-phrase some contents.

How much time did you spend on going through the checklist INCLUDING making changes in your manuscript *

2 hours.

As a result of using this checklist, do you think your manuscript has improved? *

- ○ yes
- ○ no
- ◉ Other: Some description has been changed to be more specific according to tl

Would you like to become involved in the CONSORT EHEALTH group?

This would involve for example becoming involved in participating in a workshop and writing an "Explanation and Elaboration" document

- ○ yes
- ○ no
- ◉ Other: I am overseas. If I am eligible, I would love to.

Any other comments or questions on CONSORT EHEALTH

Your answer

STOP - Save this form as PDF before you click submit

To generate a record that you filled in this form, we recommend to generate a PDF of this page (on a Mac, simply select "print" and then select "print as PDF") before you submit it.

When you submit your (revised) paper to JMIR, please upload the PDF as supplementary file.

Don't worry if some text in the textboxes is cut off, as we still have the complete information in our database. Thank you!

Final step: Click submit !

Click submit so we have your answers in our database!

SUBMIT

Never submit passwords through Google Forms.

This content is neither created nor endorsed by Google. Report Abuse - Terms of Service

Google Forms
